# Elucidating the protective mechanisms of umbilical cord mesenchymal stem cells against stenosis-induced deep venous thrombosis during pregnancy: a transcriptomic and metabolomic study

**DOI:** 10.3389/fcell.2025.1690377

**Published:** 2026-01-12

**Authors:** Junrong Zhang, Feng Sun, Jingjing Yao, Jianlin Zhang, Xirong Wu, Yunzhao Xu, Yuquan Zhang, Xi Cheng

**Affiliations:** 1 Department of Gynecology and Obstetrics, Affiliated Hospital of Nantong University, Medical School of Nantong University, Nantong, China; 2 Trauma Center, Affiliated Hospital of Nantong University, Nantong, China

**Keywords:** immune regulatory genes and metabolites, metabolomics, obstetric DVT, transcriptomics, UC-MSCs

## Abstract

**Objective:**

This study aims to integrate metabolomics and transcriptomics data to investigate the protective effects of umbilical cord mesenchymal stem cells (UC-MSCs) on obstetric deep vein thrombosis (DVT) and to elucidate the underlying molecular mechanisms.

**Methods:**

A pregnant rat model of DVT was established using the inferior vena cava (IVC) stenosis method. The protective effects of UC-MSCs on DVT and endothelial cell injury were evaluated both *in vivo* and *in vitro*. Transcriptomic and metabolomic analyses were performed to identify differentially expressed genes (DEGs) and differentially abundant metabolites (DMs) in IVC tissues from DVT rats and those treated with UC-MSCs. Correlation analysis was conducted to associate relevant metabolites and RNAs. Kyoto Encyclopedia of Genes and Genomes (KEGG) pathway enrichment analysis was applied to DEGs and DMs to identify significantly involved pathways. The mRNA–transcription factor regulatory network was constructed using Cytoscape software. Receiver operating characteristic (ROC) curves for immune regulatory genes and DEGs were generated with the R package pROC. The mMCP-counter algorithm was used to assess the distribution and abundance of immune cell subsets.

**Results:**

The rat DVT model was established using the IVC stenosis method. Administration of UC-MSCs reduced thrombus burden, promoted angiogenesis, and mitigated hydrogen peroxide-induced endothelial injury in the DVT model. Integrated transcriptomic and metabolomic analyses revealed significant correlations between four key metabolites—pyridine, nicotinamide, L-phenylalanine, and L-leucine—and 24 interacting genes. These metabolites served as critical nodes within the regulatory network. KEGG enrichment analysis indicated that pathways such as amino acid biosynthesis and phenylalanine metabolism are implicated in the therapeutic effects of UC-MSCs on pregnancy-related DVT. Notably, the hub gene Got2 was associated with amino acid biosynthesis, while both Got2 and Maoa were involved in phenylalanine metabolism. Furthermore, seven immune-regulatory genes, including Gaa and Tlr2, demonstrated significant classification performance (area under the curve [AUC] > 0.8) in ROC curve analysis.

**Conclusion:**

This study elucidates the protective mechanisms of UC-MSCs in the treatment of DVT in pregnant rats induced by the inferior vena cava stenosis model. These findings provide a scientific basis for the further evaluation and development of UC-MSCs-based therapeutic strategies for DVT during pregnancy.

## 1 Introduction

Pregnancy is a well-established risk factor for venous thromboembolism (VTE), particularly deep vein thrombosis (DVT) and pulmonary embolism (PE), which remain leading causes of maternal morbidity and mortality worldwide (Cheng et al., 2021; Nichols et al., 2020; O'Shaughnessy et al., 2020). Physiological changes during pregnancy—such as increased blood coagulability and decreased venous return due to uterine enlargement—substantially elevate the risk of thrombotic events. A systematic review of 27 original studies from 15 countries reported an incidence of DVT during pregnancy of 0.11% (95% CI: 0.10%–0.13%) (Meng et al., 2015). Despite advances in clinical guidelines and preventive strategies, the incidence of DVT among pregnant women has not declined significantly, highlighting an urgent need for improved diagnostic and therapeutic approaches.

The primary therapeutic strategies for DVT comprise anticoagulation, thrombolysis, and interventional procedures (Thaler et al., 2015; Wang et al., 2023). In the context of pregnancy, treatment selection must balance maternal and fetal safety. Prompt thrombus resolution and restoration of venous flow are essential to reduce venous hypertension, limit valvular damage, and prevent both acute and chronic complications of DVT. Nevertheless, the efficacy of these interventions is frequently limited by factors such as medication contraindications, delays in thrombolysis initiation, heightened bleeding risk, potential adverse fetal outcomes, and the development of post-thrombotic syndrome (Abdelhafez et al., 2023; Maughan et al., 2022). Consequently, the management of pregnancy-associated DVT (PA-DVT) and its related adverse outcomes remains a substantial clinical challenge.

Mesenchymal stem cells (MSCs), distinguished by their self-renewal, multipotent differentiation, and immunomodulatory capabilities, exhibit significant therapeutic potential in regenerative medicine. They have been thoroughly researched for the treatment of myocardial infarction, diabetes, and COVID-19 (Raghav and Mann, 2021; Raghav et al., 2023). The primary functional carriers of MSCs—exosomes—facilitate immune control and tissue healing, while also demonstrating considerable potential in enhancing angiogenesis and functioning as nanomedicine delivery vehicles (Raghav and Mann, 2024).

Our research group previously investigated the effects of umbilical cord-derived mesenchymal stem cells (UC-MSCs) and bone marrow-derived mesenchymal stem cells (BMMSCs) on obstetric DVT (Cheng X. et al., 2022). Significant differences were identified between the two groups regarding circulating cytokine levels and the expression of angiogenic factors and their receptors within thrombus tissue. Importantly, the UC-MSCs group exhibited a markedly higher thrombus recanalization rate than the BMMSC group. However, the mechanisms underlying the therapeutic effects of UC-MSCs on DVT during pregnancy remain to be elucidated.

Deficient thrombolysis and inadequate vascular remodeling are the principal pathogenic factors of post-thrombotic syndrome. Leveraging the established pro-angiogenic properties of UC-MSC-derived exosomes, we suggest that these exosomes may augment blood flow perfusion and accelerate thrombus recanalization in PA-DVT models by enhancing local hemodynamics and promoting angiogenesis, thereby resulting in improved clinical outcomes. This research employs a comprehensive multi-omics analytical approach to rigorously examine the concept and elucidate the molecular pathways, with the objective of objectively interpreting the functional network of UC-MSCs exosomes and identifying pivotal therapeutic biomarkers.

Integrated omics strategies—particularly the combination of metabolomic and transcriptomic profiling—have emerged as robust approaches for biomarker discovery and for elucidating disease pathogenesis at the molecular level (Qiao et al., 2021; Zhao et al., 2020). Metabolomics focuses on the comprehensive analysis of small-molecule metabolites, such as amino acids, organic acids, and lipids, thereby capturing the phenotypic endpoints of biological processes. In contrast, transcriptomics interrogates mRNA expression patterns, reflecting the underlying gene regulatory networks. Owing to their distinct yet complementary perspectives, reliance on a single omics platform often constrains the ability to fully decipher the multifactorial nature of complex diseases. The integration of transcriptomic and metabolomic data enables a more holistic characterization of disease processes by incorporating spatiotemporal context, deepening mechanistic understanding, and enhancing the precision of biomarker panels.

Metabolomics has been widely employed to identify disease biomarkers and therapeutic targets, including in the context of DVT. For instance, Li et al. (2024) utilized untargeted metabolomics to compare blood metabolite profiles between DVT rat models and healthy controls, revealing metabolites potentially involved in DVT resolution and providing novel therapeutic targets. Multiple studies have further underscored the relevance of metabolomic signatures in DVT, linking metabolic dysregulation to thrombus formation and highlighting metabolites as candidate risk biomarkers (Cao et al., 2024; Febra et al., 2024; Zhang K. et al., 2024). Concurrently, transcriptomic analyses have facilitated the identification of differentially expressed genes (DEGs) associated with DVT, thereby supporting the discovery of new therapeutic targets and the development of personalized treatment strategies (Arya et al., 2024; Fan et al., 2021; Gao et al., 2021).

Given the unclear mechanisms underlying the therapeutic effects of UC-MSCs in DVT, this study aims to systematically characterize the metabolic and transcriptomic alterations associated with UC-MSCs treatment in a PA-DVT model induced by the stenosis method in Sprague-Dawley (SD) rats. By integrating metabolomic and transcriptomic analyses, we seek to elucidate the molecular mechanisms and identify potential therapeutic targets of UC-MSCs-based interventions, thereby providing novel insights into their mode of action.

## 2 Materials and methods

### 2.1 Animals and groups

Twenty male Sprague-Dawley (SD) rats (250–300 g, 10 weeks old) and fifty female SD rats (350–400 g, 10 weeks old) were obtained from the Animal Research Facility of Nantong University. Male and female rats were housed at a 2:1 ratio under standard conditions, with *ad libitum* access to food and water and a 12-h light/dark cycle. Pregnancy was confirmed in female rats by the presence of a vaginal plug (designated as embryonic day 0.5, E0.5). Body weight of each pregnant rat was recorded daily until sacrifice. Pregnant rats were then randomly assigned to either the DVT + PBS group (*n* = 8) or the DVT + UC-MSCs group (*n* = 8). The UC-MSCs were sourced from the umbilical cord blood of healthy pregnant women, passage window used *in vivo* (P3). All experiments were conducted using cells that were free of *mycoplasma* contamination. The specific information of UC-MSCs can be supplemented in Supplementary Table 1. The grouping approach utilized a random number table method, with distinct experimenters designated to certain jobs to guarantee uniform operations. The primary outcome measure in this *in vivo* study was thrombus burden. We used GPower 3.1.9.7 software for pre-experimental power calculations, setting a two-sided test *α* = 0.05, power (1– *β*) = 0.80, and a standardized effect size d = 1.2 based on the preliminary experiment. The results showed that a sample size of *n* = 8 per group achieved ≥80% detection power. For transcriptomics and metabolomics studies, based on the RNA-seq/LC-MS differential analysis criteria, assuming a biological coefficient of variation (BCV) ≈ 0.4, expected fold change ≥1.5, and FDR <0.05, a sample size of *n* = 6 per group can guarantee approximately 80% detection power for moderate abundance features.

As previously described (Cheng et al., 2023), a DVT model was established in pregnant rats on embryonic day 11 (E10.5) using the stenosis technique applied to the lower segment of the inferior vena cava (IVC). Anaesthesia was induced in a chamber with 3%–4% isoflurane and maintained *via* a facial mask delivering 1%–2% isoflurane. Under aseptic conditions, all visible branches of the IVC below the left renal vein to the iliac vein were ligated. Subsequently, a 5–0 silk suture and an adjacent 4–0 silk suture were placed around the IVC to achieve approximately 90% stenosis, after which the 4–0 suture was removed. Before initiating *in vivo* animal tests, all rats administered human UC-MSCs through intravenous infusion received short-term pretreatment with cyclosporine A (5 μg/mL) and continued to receive low-dose cyclosporine A throughout the investigation (Girdlestone et al., 2015; Sun et al., 2017). The DVT + UC-MSCs group received three intravenous injections of umbilical cord mesenchymal stem cells (UC-MSCs; 2 × 10^6^ cells in 1 mL phosphate-buffered saline per rat) at 6 h, 1 day, and 2 days post-surgery. The DVT group received equivalent volumes of PBS at the same time points.

On the seventh postoperative day, rats were euthanized *via* carbon dioxide inhalation in a closed chamber following isoflurane anesthesia. The IVC tissue involved in thrombosis was harvested for further analysis. Additionally, tissues from the heart, liver, lung, spleen, and kidney were collected for examination. This study estimated thrombus length by directly examining the maximal diameter with a millimeter ruler in a non-stretched state post-specimen collection, whereas weight was determined using an analytical balance (after drying off the moisture). Each specimen was measured separately by two laboratory workers, and the average result was recorded to verify data correctness and repeatability. Researchers conducted separate evaluations of thrombus size and histological quantification under blinded settings to ensure data objectivity and reproducibility. All animal procedures strictly adhered to the National Institutes of Health Guide for the Care and Use of Laboratory Animals. Experimental protocols were approved by the Animal Experiment Ethics Committee of Nantong University (Approval No.: S20221231-003). Reporting of results complied with the ARRIVE guidelines.

### 2.2 Hematoxylin and eosin staining

In addition to the heart, liver, spleen, lung, and kidney, the venous thrombus generated by the stenosis method and placenta were fixed in 4% paraformaldehyde for 24 h. Subsequently, all tissues were dehydrated through graded ethanol concentrations, embedded in paraffin, and sectioned at a thickness of 4–6 μm. The sections were then stained with haematoxylin and eosin (H&E) according to standard protocols.

### 2.3 Immunofluorescence staining

Tissues were fixed in 4% paraformaldehyde and then incubated with 5% bovine serum albumin to block nonspecific IgG binding. Subsequently, cells were incubated at 4 °C with anti-CD34 antibody (144860-1-AP, Proteintech), or anti-CD31 antibody (66065-2-Ig, Proteintech), followed by incubation with a Cy3-conjugated secondary antibody (SA00009-2, Proteintech) for 1 h at room temperature. Fluorescence images were acquired using a fluorescence microscope.

Concerning “microvessel density counts,” we predominantly utilized the Weidner method for measurement, adhering to the streamlined protocol outlined in the reference literature (Hassaf-Arreola et al., 2022). The approach encompasses: Initially, assess CD34 or CD31-stained sections using low-power microscopy to locate regions abundant in microvessels, referred to as “hotspot” areas. Three fields exhibiting distinct staining and good background contrast are chosen for photographic documentation under high-power microscopy. Two pathologists, oblivious to the experimental group allocations, independently conduct microvessel density assessments to mitigate subjectivity. In each segment, both pathologists individually pick and quantify fields, with the final density value determined as the average of their readings.

### 2.4 Immunohistochemistry

Tissue samples were fixed in 4% paraformaldehyde for 24 h, followed by dehydration through a graded ethanol series and paraffin embedding. Sections were cut at a thickness of 4–6 μm for immunohistochemical analysis. After dewaxing, rehydration, and antigen retrieval, the sections were incubated with anti-CD34 or anti-CD31 antibody (1:1000, Proteintech). Subsequently, a biotin-conjugated secondary antibody was applied for nuclear staining. Images were acquired using an Olympus optical microscope.

### 2.5 Cell culture and treatment

HUVECs were kindly provided by Associate Professor Yan Xue (Nantong University, China). Cells were cultured at 37 °C in a humidified incubator with 5% CO_2_ in endothelial cell medium (ECM) supplemented with 10% fetal bovine serum (FBS) and 1% penicillin/streptomycin (Sciencell, USA). HUVECs were passaged at a 1:3 ratio upon reaching confluence following trypsinization. Only cells from passages 3 to 8 were used for experiments. To model vascular injury associated with DVT, HUVECs were treated with 200 μmol/L hydrogen peroxide (H_2_O_2_; Hengjian Pharmaceutical, Guangdong, China), as described in previous studies using the CCK-8 assay (Cheng et al., 2023).

To assess the effects of MAOA, we categorized the cells into four groups: sham control, H_2_O_2_ model, UC-CM treatment, and UC-CM in conjunction with MAOA inhibitor. We administered clorgyline (1 μM), a sole MAOA inhibitor, adhering to doses and treatment durations established in previous investigations to maintain experimental consistency (Li et al., 2017; Wu et al., 2014).

To assess GOT2 functionality, we categorized cells into three groups: sham control, DVT model, and DVT with GOT2 inhibitor therapy. At present, there is a paucity of highly selective small-molecule inhibitors that selectively target GOT2. To tackle this issue, we utilized recognized research methodologies and administered the broad-spectrum transaminase inhibitor amino oxime acid (AOA, 750 μM) to proficiently limit GOT2 activity, thereby evaluating its possible function in metabolic control (Lee et al., 2020; Mei et al., 2021).

### 2.6 Conditioned medium preparation

FBS was omitted during the 48-h incubation in DMEM/F12 medium. To obtain umbilical cord-conditioned medium (UC-CM), the supernatant was collected and concentrated using an Amicon Ultra-15 centrifugal filter unit (3 kDa cutoff, UFC900396; Millipore, Darmstadt, Germany) by centrifugation at 4,000 rpm for 30 min. The resulting UC-CM was stored at −80 °C for subsequent use.

### 2.7 Scratch wound healing assay

HUVECs (2 × 10^5^ cells/well) were seeded in 12-well plates (*n* = 3 per group) and cultured in endothelial cell medium (ECM) for 12 h. After cell attachment, the medium was replaced with ECM containing 200 μmol/L H_2_O_2_ for an additional 12 h. Linear scratches were then created on the cell monolayer using a sterile 10-μL pipette tip, followed by washing with PBS. Subsequently, the cells were incubated in serum-free ECM, UC-CM, or ECM for 12 h. Images of the wound area were captured at 0 and 12 h post-scratch. The cell migration rate was calculated as the ratio of the wound closure area at 12 h to the initial wound area at 0 h.

### 2.8 Transwell migration and invasion assay

Impaired HUVECs were seeded at a density of 5 × 10^4^ cells per well in 500 μL of ECM in the upper chambers of 24-well Transwell plates with 8-μm pore size inserts (BD Biosciences, CA, USA). For the invasion assay, 200 μL of Matrigel (1 mg/mL) was applied to the upper surface of the inserts. The lower chambers were filled with either UC-CM or ECM. After incubation for 21 h (migration assay) or 36 h (invasion assay), non-migratory cells remaining on the upper surface of the membrane were removed with a sterile cotton swab. Migrated cells on the lower surface were stained with 0.5% crystal violet and imaged using a fluorescence microscope (Olympus BX51). For quantification, five randomly selected fields per group were analyzed.

### 2.9 Western blotting

Proteins were extracted from thrombosed IVC tissues and HUVECs using radioimmunoprecipitation assay (RIPA) lysis buffer supplemented with 1% phenylmethanesulfonyl fluoride, 1% protease inhibitor, and 1% protease inhibitor cocktail (KeyGen Biotech, Nanjing, China). The protein samples were separated by 10% sodium dodecyl sulfate-polyacrylamide gel electrophoresis (SDS-PAGE) and transferred onto polyvinylidene difluoride (PVDF) membranes. Membranes were blocked with 5% skim milk at room temperature for 2 h to prevent non-specific binding. Subsequently, the membranes were incubated overnight at 4 °C with primary antibodies against Got2 (1:8000; Proteintech), Maoa (1:1000; Proteintech), and β-actin (1:1000; Abcam). After washing, membranes were incubated with goat anti-rabbit secondary antibody (1:2000; Abcam). Protein bands were visualized using the Tanon 2500 Gel Imaging System (Tanon, Shanghai, China).

### 2.10 RT-qPCR assay

Total RNA was extracted from thrombosed IVC tissues using TRIzol reagent (Invitrogen, Waltham, USA) according to the manufacturer’s instructions. Complementary DNA (cDNA) was synthesized with the HiScript™ II Q RT SuperMix for qPCR (+gDNA wiper) kit (Vazyme, Nanjing, China). Quantitative real-time PCR (RT-qPCR) was conducted in a 20-μL reaction volume using ChamQ Universal SYBR qPCR Master Mix (Vazyme, Nanjing, China) on a LightCycler 480 system (Roche), following the manufacturer’s protocol. The primer sequences for Maoa, Got2, and HPRT1 were as follows: Maoa in rat (F: CCC​GAG​TCC​AAG​GAT​GTT​CC; R: TGA​TCT​TGA​GCA​GAC​CAG​GC), Got2 in rat (F: GCG​GCT​TTG​ACT​TCT​CTG​GA; R: AAA​GCC​TTG​GTA​GGC​CAT​GT), HPRT1 in rat (F:TGTTTGTGTCATCAGCGAAAGTG; R: ATT​CAA​CTT​GCC​GCT​GTC​TTT​TA). Relative gene expression levels were calculated using the 2^–ΔΔCT^ method, with HPRT1 as the internal control. The stability of HPRT1 as a reference gene was validated using the geNorm algorithm.

### 2.11 ROS detection

The production of intracellular ROS was assessed using the ROS fluorescent probe CM-H2DCFDA (Beyotime, China). In summary, following the treatment of cells, the cells were loaded with the CM-H2DCFDA probe for 30 min. Subsequently, the cells were washed once with PBS and detected using a fluorescence microscope.

### 2.12 RNA sequencing

Total cDNA synthesized from mRNA isolated from thrombosed tissues in the DVT + PBS and DVT + UC-MSCs groups (*n* = 6 per group) was subjected to high-throughput sequencing. This approach enabled comprehensive and rapid acquisition of mRNA sequence information from thrombus tissues. RNA extraction, sample quality assessment, library construction, library quality control, and sequencing were performed by Applied Protein Technology Co. Ltd. (Shanghai, China).

### 2.13 Non-targeted metabolomics profiling

Following thrombus sample extraction, analyses were performed using a liquid chromatography-mass spectrometry (LC-MS) platform. A 2 μL injection volume was utilized, with the autosampler maintained at 4 °C. Primary and secondary mass spectrometry data were acquired using an Orbitrap Exploris 120 mass spectrometer (Xcalibur 4.4, Thermo Fisher Scientific). All experiments were conducted by Beijing Allwegene Technology Co., Ltd. (Beijing, China). See the Supplementary material (2.12 Non-targeted metabolomics profiling part) for the specific operation process.

### 2.14 Difference analysis

Samples were categorized into DVT and UC-MSCs groups according to dataset grouping. Differential gene expression analysis between thrombosed and treated samples was conducted using the DESeq2 package (version 1.42.0) in R (Love et al., 2014). The resulting differentially expressed genes were visualized with a volcano plot generated by the ggplot2 package (version 3.4.4).

Metabolomics data were processed and analyzed using the MetaboAnalystR package. Initially, a data object was established for statistical analysis of peak intensity values. Data completeness was assessed, and missing values were imputed with the minimum observed value. Variables were filtered by applying a relative standard deviation threshold of 25% and using the interquartile range to prepare the preprocessed dataset. Data normalization was performed using sum normalization, log transformation, and mean centering within the package. Differential analysis included fold-change (FC) analysis (threshold: 1) and t-test (significance level: 0.05) with false discovery rate correction. Subsequently, orthogonal partial least squares discriminant analysis (OPLS-DA) was performed to evaluate the classification performance of the dataset. In OPLS-DA, variable importance in projection (VIP) scores were used to assess each variable’s contribution to group discrimination. Metabolites with VIP scores greater than 1 and *p*-values less than 0.05 were considered significant and selected for further analysis.

Additionally, immune regulation-related genes (IRGs) were retrieved from the GeneCards database, a comprehensive resource for human gene information (O'Shaughnessy et al., 2020; Pang et al., 2024). The keyword “Immunomodulation” was used for the search, and genes with correlation scores exceeding 2 were identified as IRGs. Detailed information is provided in Supplementary Table 2.

### 2.15 Correlation analysis

In this study, correlation analysis was conducted to assess the relationships between metabolite and gene expression data. To ensure accurate sample matching, column names of the metabolite and gene expression datasets were standardized. Subsequently, the weighted gene co-expression network analysis (WGCNA) package (v1.72-5) was employed to calculate Pearson correlation coefficients and corresponding p-values for each metabolite–gene pair across matched samples. Highly correlated pairs were defined as those with correlation coefficients greater than 0.8 and *p*-values less than 0.05. Based on the log2 fold-change (FC) values of genes and metabolites, results were categorized into nine quadrants to delineate distinct patterns of change. Visualization was performed using the ggplot2 package, generating a nine-quadrant plot to illustrate the associations between metabolites and gene expression. This study utilized only correlation calculation functions (e.g., cor and corPvalueStudent) from the WGCNA package to effectively compute Pearson correlation coefficients and their significance tests for extensive expression matrices.

For the identified significant metabolite–gene pairs, network analysis was carried out using the MetaboAnalystR package. Genes and metabolites were subsequently mapped to KEGG database, and gene–metabolite interaction networks were constructed based on KEGG pathway information.

### 2.16 KEGG pathway enrichment analysis

KEGG is a widely used database that provides comprehensive information on genomes, biological pathways, diseases, and drugs. In this study, functional annotation and pathway enrichment analysis of genes and metabolites were performed using the clusterProfiler R package (v4.10.0) based on the KEGG database. Pathways with a *p*-value <0.05 and a q-value <0.2 were considered significantly enriched, with the q-value used to control the false discovery rate. Subsequently, overlapping metabolic and gene enrichment pathways were identified and selected for further analysis.

### 2.17 Construction of regulatory network

Transcription factors (TFs) regulate gene expression primarily by binding to specific genomic regions and influencing transcriptional activity. In this study, TFs and their regulatory effects on target genes were identified using the ChIPBase database (http://rna.sysu.edu.cn/chipbase/). The resulting mRNA–TF regulatory network was visualized using Cytoscape software. Additionally, microRNAs (miRNAs) are key post-transcriptional regulators that modulate a wide range of target genes, often enabling the same gene to be regulated by multiple miRNAs. To investigate the interactions between genes and miRNAs, gene-associated miRNAs were retrieved from the StarBase v3.0 database (https://starbase.sysu.edu.cn/), and the mRNA–miRNA regulatory network was constructed and visualized using Cytoscape. See Supplementary Table 3 for specific information.

### 2.18 Immune-related gene ROC analysis

To evaluate the discriminatory ability of immune-related genes for distinguishing between normal and treatment groups, receiver operating characteristic (ROC) curve analysis was performed using the pROC package in R. Area under the curve (AUC) values were calculated for each gene, with AUC > 0.5 indicating discriminatory ability. To assess the statistical precision of AUC estimates, we employed stratified bootstrap resampling with 2,000 iterations. In each bootstrap iteration, samples were randomly drawn with replacement from each class (normal and treatment) while maintaining the original class proportions. The 95% confidence intervals were calculated using the percentile method (2.5th and 97.5th percentiles of the bootstrap AUC distribution). This approach provides robust uncertainty estimates and accounts for sampling variability without requiring data splitting.

### 2.19 Immune infiltration analysis (mMCP-counter) and consistency cluster analysis

mMCP-counter, a deconvolution method specifically designed for mouse and rat transcriptomic data, was utilized to estimate the abundance of immune cell types across samples. This algorithm quantifies 10 immune cell types including T cells, CD8 T cells, cytotoxic lymphocytes, B lineage, NK cells, monocytic lineage, myeloid dendritic cells, neutrophils, endothelial cells, and fibroblasts. The resulting immune cell infiltration matrix was used to generate bar plots to visualize and analyze the proportions of different immune cell populations.

Consensus clustering, implemented *via* the R package ConsensusClusterPlus (v1.62.0), was applied to identify distinct subtypes among treated samples based on the expression of two hub genes (Got2 and Maoa) (Lock and Dunson, 2013). This method employs repeated subsampling and resampling to assess cluster stability and optimize parameter selection (Wilkerson and Hayes, 2010). Specifically, the number of clusters (k) was set between 2 and 3, with 80% of the samples randomly selected and resampled 1,000 times using the k-means algorithm (ClusterAlg = “km”) and Euclidean distance metric (distance = “euclidean”).

### 2.20 Statistical analysis

All data processing and analyses were conducted using R software (version 4.2.0). Continuous variables are expressed as mean ± standard deviation. Comparisons between two groups were performed using the Wilcoxon rank-sum test. Unless otherwise specified, correlations between different molecules were assessed using Spearman’s correlation analysis. Statistical significance was defined as an adjusted *p*-value (adj. *p*) less than 0.05.

## 3 Results

### 3.1 UC-MSCs promote angiogenesis and may improve pregnancy outcomes in pregnant rats with DVT

**FIGURE 1:**
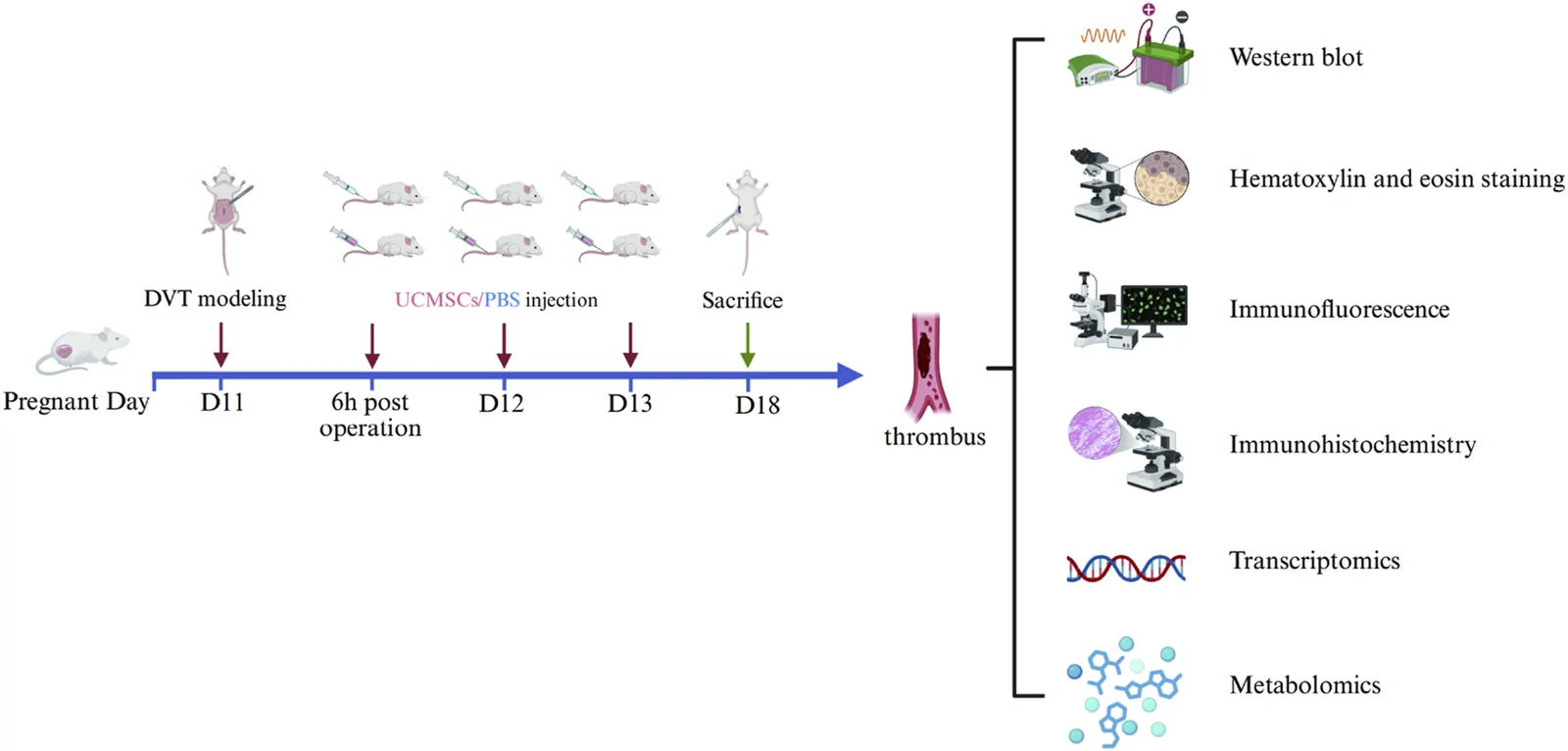
Experimental design schematic.

The experimental design schematic is presented in Figure 1.

**FIGURE 2:**
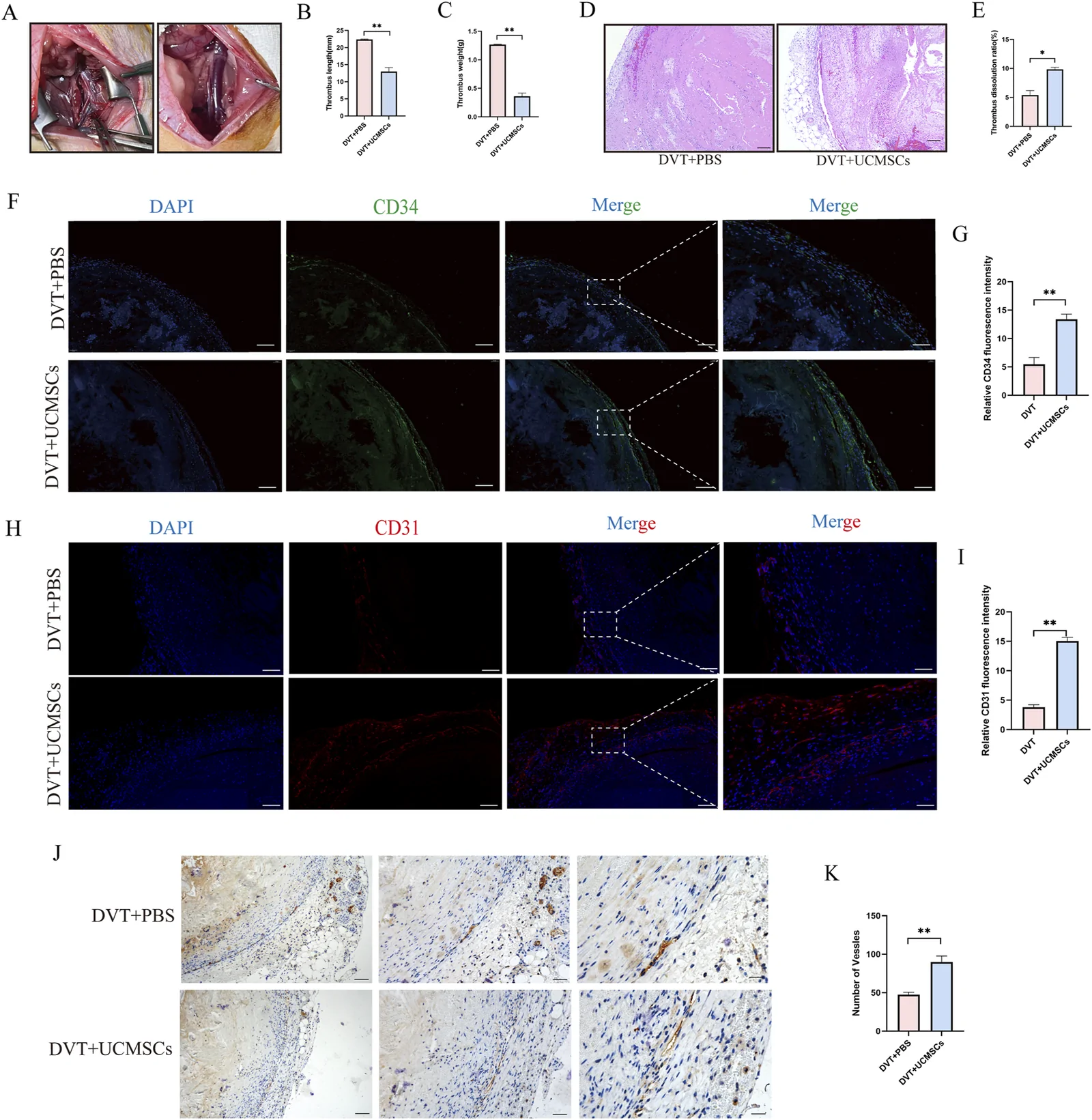
Three successive injections of UC-MSCss markedly decreased both the length and weight of thrombi in pregnant mice with DVT, concurrently facilitating thrombus recanalisation. **(A)** DVT model in pregnant Sprague-Dawley rats established using the stenosis approach; **(B)** Statistical analysis results of thrombus length; **(C)** Statistical analysis results of thrombus weight; **(D)** HE staining of thrombus. Scale bars: 200 μm; **(E)** Statistical analysis results of thrombus dissolution ratio across several groupings; **(F,G)** Representative IF images of CD34 (green) of thrombus tissue, nuclei are stained blue (scale bar = 100 μm). Relative protein expression levels were calculated; **(H,I)** Representative IF images of CD31 (red) of thrombus tissue, nuclei are stained blue (scale bar = 100 μm). Relative protein expression levels were calculated; **(J,K)** CD34 immunohistochemical staining and numbers of positive vessles. Scale bars: 200 μm (*n* = 3). Data are presented as the mean ± SEM. (*p < 0.05, **p < 0.01, ***p < 0.001, ns, no significance).

Figure 2A depicts the establishment of the SD pregnant rat DVT model using the stenosis technique. Compared with the DVT + PBS group, the UC-MSCs-treated groups exhibited significantly reduced thrombus length and weight (*p* < 0.05; Figures 2B,C). HE staining revealed extensive luminal occlusion with minimal thrombus disintegration in the DVT + PBS group, whereas the UC-MSCs group demonstrated marked improvement in vessel wall patency (Figures 2D,E). To evaluate angiogenesis within the thrombus, CD34 and CD31 Immunofluorescence staining was performed (Figures 2F–I). Meanwhile, CD34 immunohistochemical was also used to detect the number of new blood vessels (Figures 2J,K). The number of CD34-positive vessels was significantly higher in the UC-MSCs group than in the DVT + PBS group (*p* < 0.01). These results indicate that UC-MSCs markedly promote neovascularization and effectively reduce thrombus size in the rat DVT model.

**FIGURE 3:**
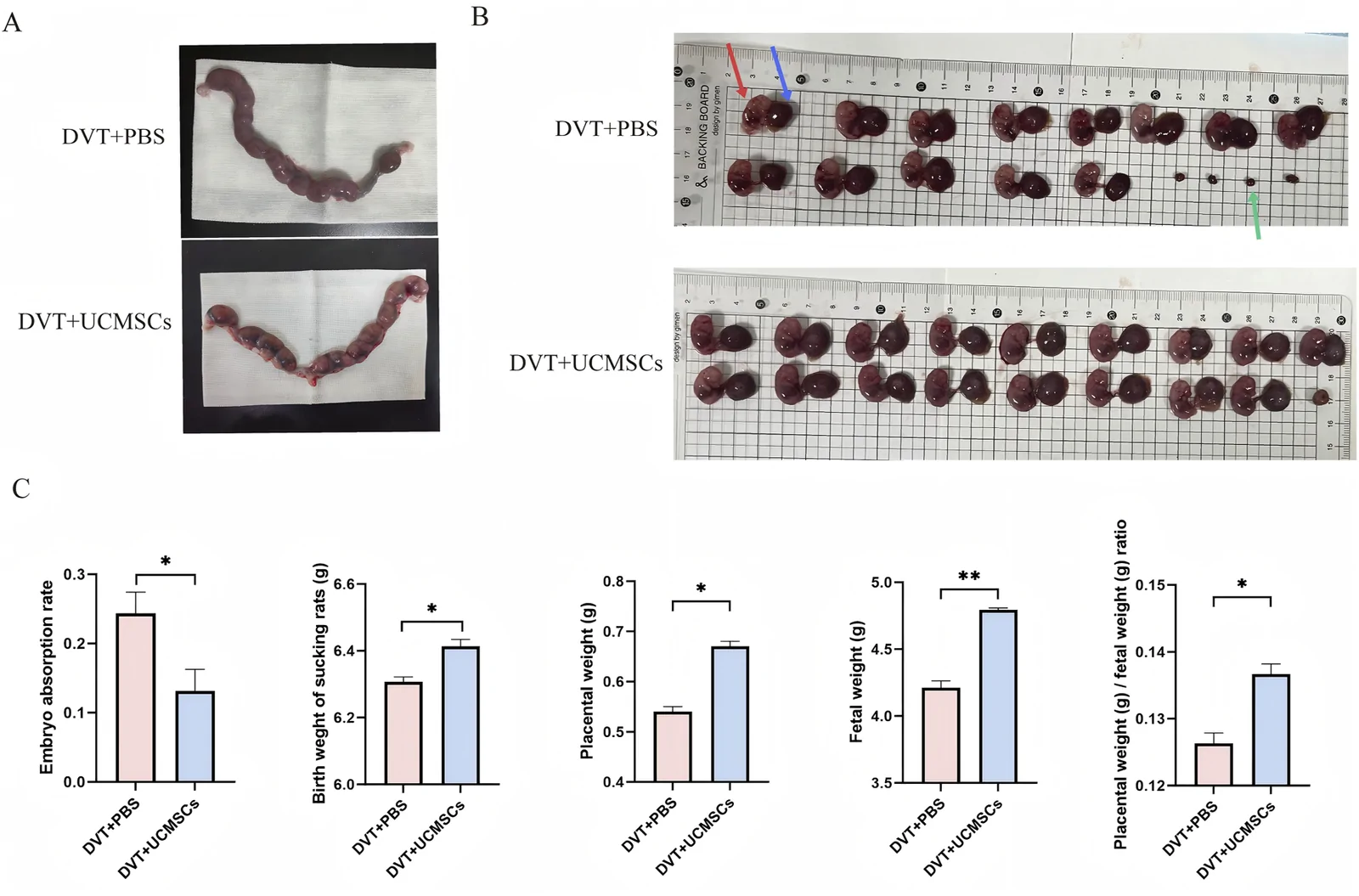
UC-MSCs have the ability to improve pregnancy outcomes in pregnant DVT rats *in vivo*. **(A)** Examples of the uterine horn that show the rescue impact of UC-MSCss in DVT pregnancies and increased fetal loss in DVT models; **(B)** Typical anatomical images of placentas and embryos from different groups; the red arrow denotes the fetal rat and the blue arrow represents placenta, whereas the green arrow highlights the residual placental tissue following embryo absorption. **(C)** The rates of embryo resorption, birth weight of suckling rats, placental weight, fetal weight, and the ratio of placental weight to birth weight in the two groups. *n* = 6 per group. Data are presented as the mean ± SEM. *p < 0.05, **p < 0.01, ***p < 0.001, NS, no significance.

Additionally, the impact of UC-MSCs on pregnancy outcomes in rats with PA-DVT was evaluated by comparing embryonic resorption rates between groups. The DVT + PBS group exhibited a significantly higher embryonic resorption rate than the UC-MSCs-treated group (Figures 3A,B). Furthermore, UC-MSCs transplantation markedly reduced the embryonic resorption rate and significantly increased both average embryonic and placental weights compared with the DVT + PBS group (Figure 3). Analysis of neonatal birth weights revealed that live pups in the UC-MSCs-treated group had significantly greater birth weights than those in the DVT + PBS group (Figure 3C), indicating that UC-MSCs treatment improves pregnancy outcomes in this model.

### 3.2 *In vitro*, UC-MSCs significantly promoted the proliferation, migration, and invasion of HUVECs

**FIGURE 4:**
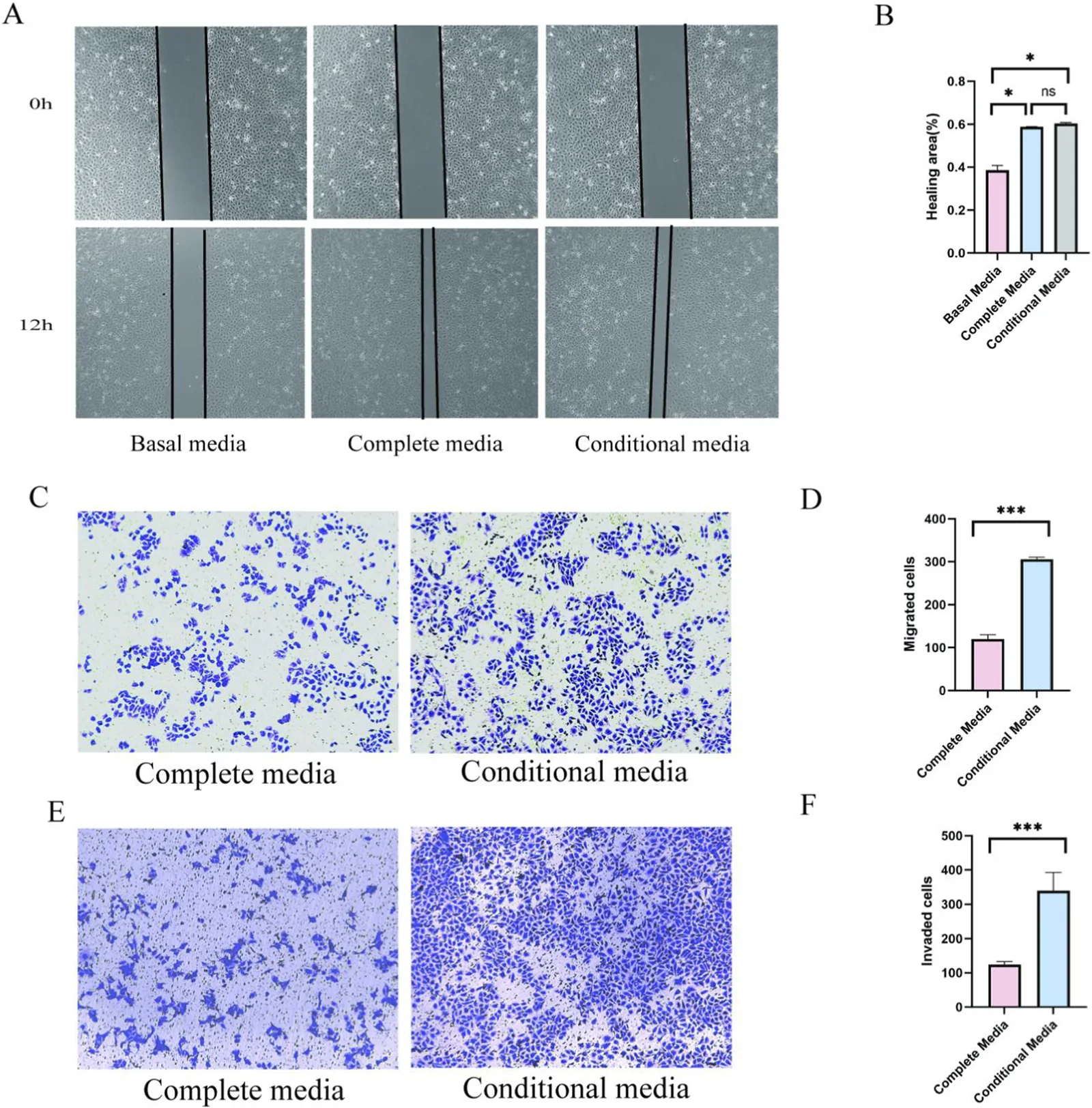
UC-CM can repair endothelial cell damage induced by H_2_O_2_. **(A)** The effect of UC-CM on the healing of damaged endothelial cells was assessed using the scratch assay. Scale bars: 100 µm; **(B)** Statistical analysis results of healing area; **(C, D)** Numbers of crystal violet-stained migrated cells. Scale bars: 100 µm; **(E, F)** Numbers of crystal violet-stained invased cells. Scale bars: 100 µm. (*n* = 3 biological replicates). Data are presented as the mean ± SEM. (*p < 0.05, **p < 0.01, ***p < 0.001, ns, no significance).

The effects of UC-CM on the proliferation, migration, and invasion of endothelial cells subjected to H_2_O_2_-induced injury were further evaluated *in vitro*. Based on preliminary findings, treatment with 200 μmol/L H_2_O_2_ for 12 h reduced cell viability to approximately 50% of the control group; therefore, this concentration and duration were selected for subsequent experiments (Cheng et al., 2023). In the scratch wound assay, UC-CM significantly promoted the migration of injured HUVECs, achieving migratory capacity comparable to or exceeding that of cells maintained in complete or basal media, respectively (Figures 4A,B). Similarly, the transwell migration assay revealed that UC-CM exerted a more pronounced stimulatory effect on migration than complete culture medium (Figures 4C,D). Moreover, the transwell invasion assay demonstrated that UC-CM markedly enhanced the invasive ability of damaged HUVECs compared with those cultured in complete medium for 36 h (Figures 4E,F). *In vitro* WB assays revealed that UC-CM markedly increased the angiogenesis-related components in compromised HUVECs (Figure 7G). The results of *in vitro* experiments were biological replicates for three times (Supplementary material - Repeated independent experiments for *in vitro*). Collectively, these results indicate that UC-CM substantially mitigates oxidative damage and enhances the migration, invasion, and survival of injured endothelial cells.

### 3.3 Differential analysis

**FIGURE 5:**
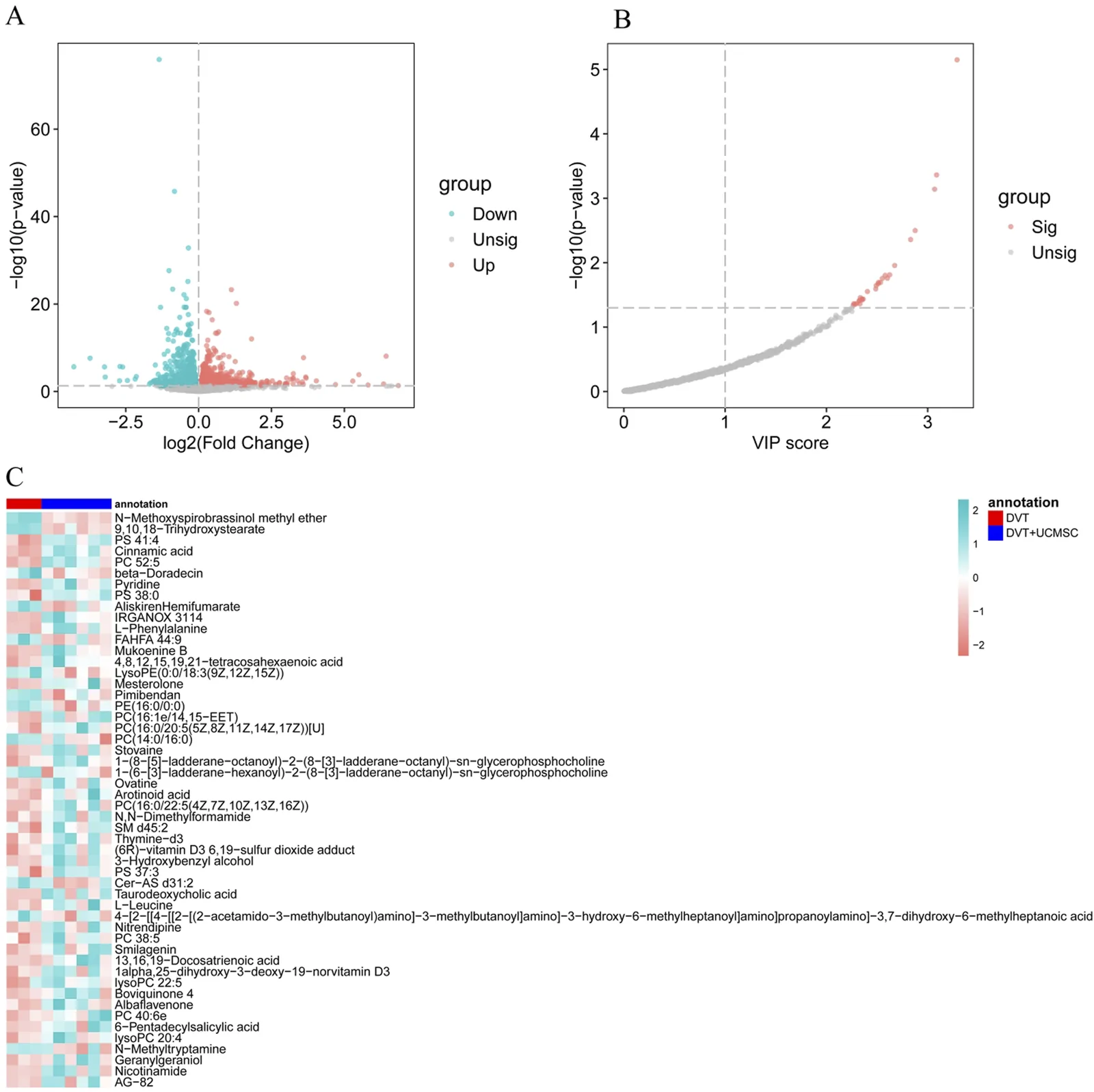
Differential analysis of DEGs and DMs. **(A)** Volcano plot of the DEGs analysis of DVT and UC-MSCss samples in the transcriptome dataset; **(B)** VIP scatter plot in the metabolome dataset; **(C)** Heat map of important metabolites with VIP greater than 1 in the metabolome dataset.

Supplementary Figure 1 presents the experimental workflow. The transcriptome dataset was categorized into DVT and UC-MSCs groups. To identify DEGs between these groups, differential expression analysis was conducted using the DESeq2 R package. A total of 2,058 DEGs with *p* < 0.05 were identified. The results are visualized in a volcano plot (Figure 5A). We then applied *p.adj*<0.05 to further screen for DEGs. The results showed that the number of DEGs decreased by 4 after using p.adj values, as illustrated in the volcano plot in the Supplementary Figure 2. Subsequently, orthogonal partial least squares discriminant analysis (OPLS-DA) was applied to the metabolomic data to identify key metabolites with VIP scores greater than 1 (Figure 5B). We also conducted a comprehensive permutation tests of the OPLS-DA model and a OPLS-DA score plot, incorporating supplementary R2Y and Q2 statistical metrics, along with 200 permutation test results (Supplementary Figures 3A,B). A heatmap of these important metabolites was generated using the pheatmap R package (Figure 5C).

### 3.4 Correlation analysis and enrichment analysis

**FIGURE 6:**
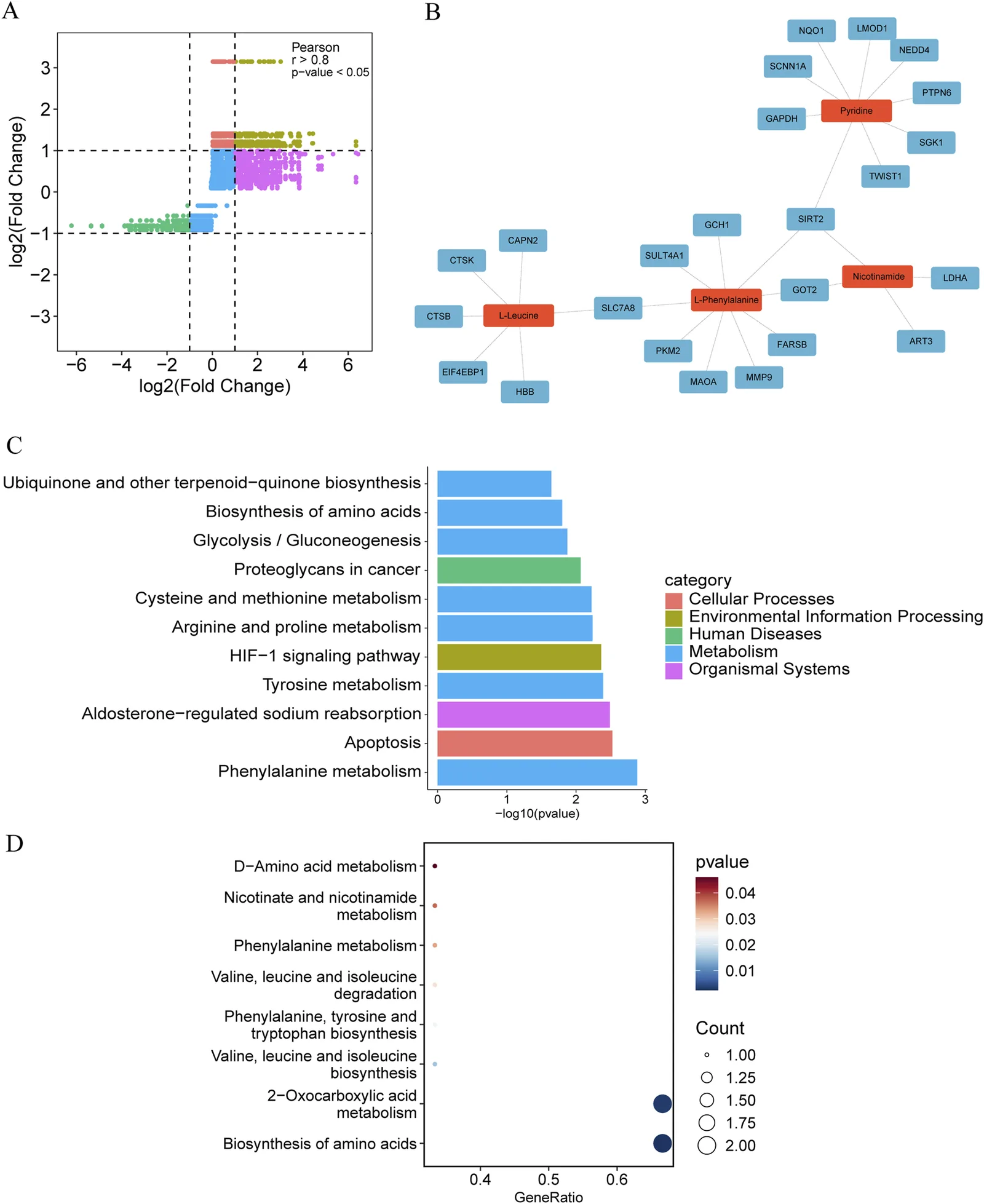
Correlation analysis and enrichment analysis. **(A)** The gene-metabolite nine-quadrant plot, with the abscissa representing the log2FC of the gene and the ordinate representing the log2FC of the metabolite. The different colors in the plot distinguish points in different quadrants, representing different patterns of change; **(B)** High correlation gene-metabolite pair interaction network, where red is the metabolite and blue is the gene; **(C)** Bar chart of KEGG pathway enrichment analysis results of highly correlated genes; **(D)** Bubble chart display of pathway (KEGG) enrichment analysis results of highly correlated metabolites.

In Figure 6A, the x-axis represents gene expression changes (over-expression or downregulation), while the y-axis depicts metabolite regulation (up- or downregulation). Each dot of varying color on the plot indicates a specific gene–metabolite pair. Spearman rank correlation analysis was performed to characterize the associations between DEGs and DMs. A positive correlation coefficient (>0) indicates that increased gene expression is associated with elevated metabolite levels, whereas a negative coefficient (<0) suggests that higher gene expression corresponds to reduced metabolite concentrations. A coefficient of zero denotes no association between the gene and metabolite. For example, the upper right quadrant of Figure 6A corresponds to gene–metabolite pairs in which both gene expression and metabolite abundance are increased (positive log2FC values). Gene–metabolite pairs with a correlation coefficient greater than 0.8 and a *p*-value less than 0.05 were identified as highly correlated (Figure 6A).

To elucidate the interactions between genes and metabolites within key pathways, we constructed an interaction network model to visually represent the relationships between DEGs and DMs. This visualization enables clear identification of relevant genes and metabolites and intuitively demonstrates their potential interaction networks. Subsequently, gene–metabolite pairs with strong correlations were analyzed based on the KEGG database. The analysis revealed interactions involving four metabolites and 24 genes (Figure 6B). Notably, metabolites such as pyridine, nicotinamide, L-phenylalanine, and L-leucine emerged as central nodes, interacting with multiple genes. These findings indicate that these metabolites play pivotal roles in the investigated metabolic pathways and may have significant implications for therapeutic strategies.

In metabolomics research, the qualitative accuracy of metabolites—particularly key metabolites—is of critical importance. Currently, metabolite identification confidence is generally categorized into five levels: (1) Level 1: Confirmed structure; (2) Level 2: Probable structure; (3) Level 3: Tentative candidates; (4) Level 4: Unequivocal molecular formula; and (5) Level 5: Exact mass of interest (Schymanski et al., 2014). The detailed criteria are illustrated in Supplementary Figure 4. In this study, all metabolites were annotated according to standards to ensure high qualitative accuracy. By meticulously identifying difference metabolites, we discerned several crucial metabolites. Among them, pyridine, L-leucine, L-phenylalanine, and nicotinamide were identified as marker metabolites with confirmed structures (Level 1). To be specific, the primary and secondary mass spectra were first matched with mass tolerances of 5 ppm and 10 ppm, respectively. The actual precursor mass errors were 4.91, 4.00, 1.43, and 1.36 ppm, and the MS/MS similarity scores were all above 0.7 (0.998, 0.730, 0.795, and 0.755, respectively). To further verify, these results were further validated using authentic standards of the four compounds. As shown in Supplementary Figure 5, the retention times (RT) of the standard MS1 spectra—0.85, 1.46, 2.25, and 1.11 min—were consistent with those observed in the sample. Moreover, Supplementary Figure 6 presents a comparison of the experimental MS/MS spectra with the corresponding standard spectra, demonstrating excellent spectral consistency. Consequently, the ions at m/z 80.0499 (*t*_R_ = 0.88 min), m/z 132.1013 (*t*_R_ = 1.48 min), m/z 166.0860 (*t*_R_ = 2.30 min), and m/z 123.0551 (*t*_R_ = 1.08 min) were confidently identified as pyridine, L-leucine, L-phenylalanine, and nicotinamide, respectively. Identification of other metabolites followed the same principles.

KEGG enrichment analysis was conducted for both metabolites and genes. The results demonstrated that differentially expressed genes were predominantly enriched in pathways such as phenylalanine metabolism, apoptosis, aldosterone-regulated sodium reabsorption, tyrosine metabolism, HIF-1 signaling, arginine and proline metabolism, cysteine and methionine metabolism, proteoglycans in cancer, glycolysis/gluconeogenesis, biosynthesis of amino acids, and ubiquinone and other terpenoid-quinone biosynthesis (Figure 6C). Differential metabolites were mainly enriched in biosynthesis of amino acids, 2-oxocarboxylic acid metabolism, valine, leucine and isoleucine biosynthesis, phenylalanine, tyrosine and tryptophan biosynthesis, valine, leucine and isoleucine degradation, phenylalanine metabolism, nicotinate and nicotinamide metabolism, and D-amino acid metabolism (Figure 6D). Notably, both differentially expressed genes and metabolites were commonly involved in the biosynthesis of amino acids and phenylalanine metabolism pathways.

### 3.5 Construction of the regulatory network

**FIGURE 7:**
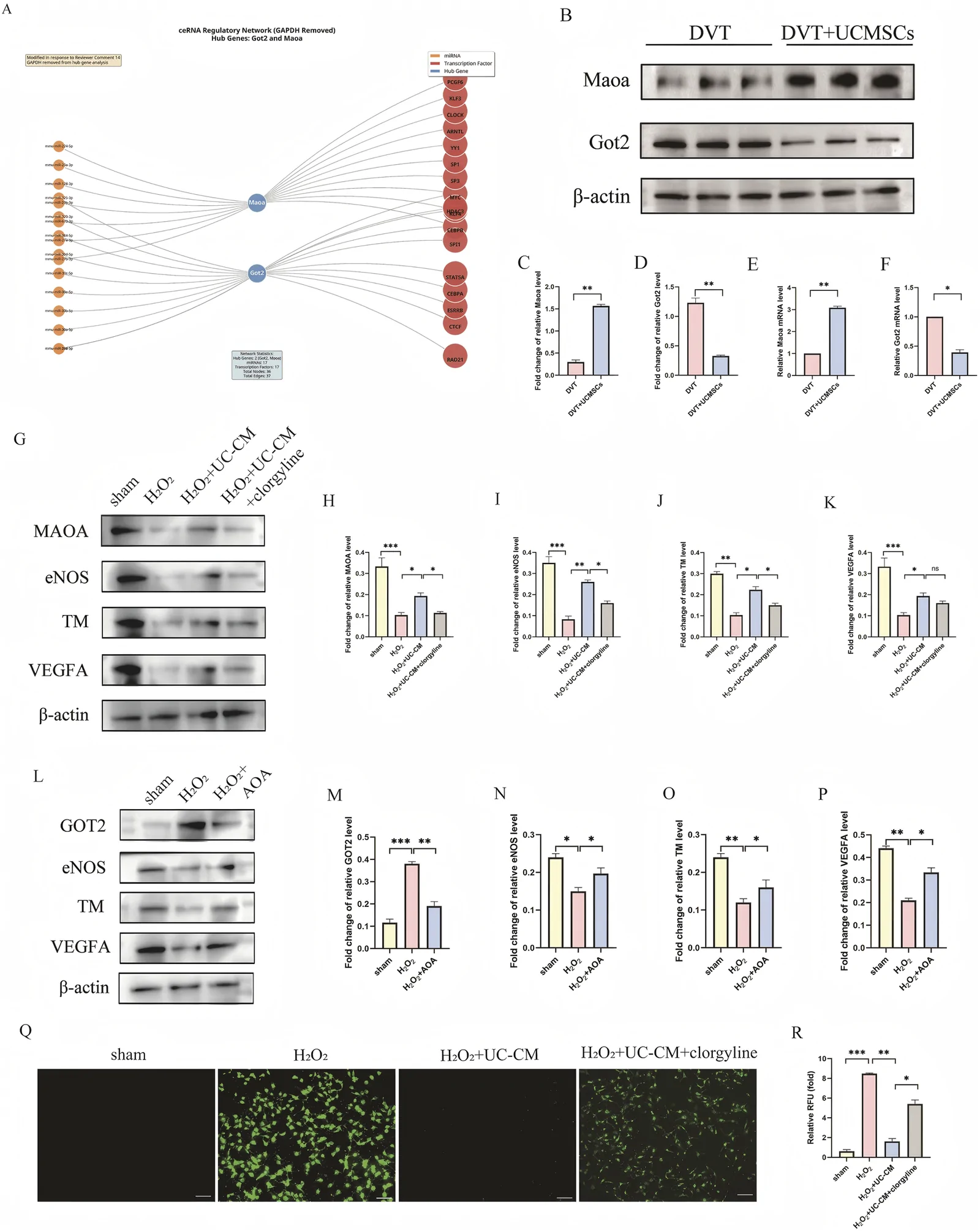
MiRNA-mRNA-TF Regulatory Network and causality assays of hub genes. **(A)** Construction of the miRNA-mRNA-TF Regulatory Network of hub genes. TF, transcription factor; orange represents miRNA, blue represents mRNA (hub gene), and red represents TF. **(B–D)** Representative Western blot images of Maoa, Got2 from thrombosed IVC tissues. **(E,F)** qRT-PCR analysis of Maoa, Got2 expression from thrombosed IVC tissues. **(G–K)** Representative Western blot images of MAOA, eNOS, TM and VEGFA and their quantitative results using MAOA inhibitor (clorgyline) in endothelial cell damage induced by H_2_O_2_ (*n* = 3). **(L–P)** Representative Western blot images of GOT2, eNOS, TM and VEGFA and their quantitative results using GOT2 inhibitor (AOA) in endothelial cell damage induced by H_2_O_2_ (*n* = 3). **(Q,R)** *In vitro*,the production of ROS was assessed using the CM-H2DCFDA probe. Data are presented as the mean ± SEM. (*p < 0.05, **p < 0.01, ***p < 0.001, ns, no significance).

First, miRNAs targeting the hub genes Got2 and Maoa were identified using the StarBase database. mRNA–miRNA pairs that were supported by multiple databases and exhibited high interaction counts were selected to construct the mRNA–miRNA regulatory network. Subsequently, TFs associated with the hub genes were identified through the ChIPBase database. mRNA–TF pairs with a greater number of binding sites were selected to establish the mRNA–TF regulatory network. Detailed information is provided in Supplementary Table 3. All networks were visualized using Cytoscape software (Figure 7A). Hub genes were identified as those related with the aforementioned common pathways: Got2 for amino acid biosynthesis, and both Got2 and Maoa for phenylalanine metabolism. GAPDH was excluded from hub gene analysis to avoid the logical inconsistency of using it as both a study target and a reference gene. To validate these hub genes, pregnant SD rats were divided into DVT and DVT + UC-MSCs groups for experimental verification. Western blot and qRT-PCR analysis showed that, compared with the DVT group, Maoa protein expression was increased and Got2 expression was decreased in the UC-MSCs group (Figures 7B–F).

To elucidate the precise functions of these two molecules (MAOA and Got2) in endothelial activity, we performed systematic pharmacological intervention experiments in HUVECs. We examined the impact of these therapies on critical thrombosis-related and angiogenesis-related variables (eNOS, TM, VEGF-A) using wb analysis. The results indicated that the addition of the MAOA inhibitor clofibrate to the UC-CM group dramatically reduced the production of the anti-thrombotic factors eNOS and thrombomodulin (TM), along with the pro-angiogenic factor VEGFA (Figures 7G–K). In contrast, in H_2_O_2_ model, the inhibition of GOT2 activity by AOA resulted in a substantial overexpression of eNOS, TM, and VEGFA (Figures 7L–P). Additionally,we detected the differences in ROS expression in the MAOA group.The results showed that the UC-MSCs treatment group reduced H_2_O_2_-induced ROS expression. However, after adding the MAOA inhibitor clorgyline to the UC-MSCs group, ROS production significantly increased (Figures 7Q,R).

### 3.6 Verification of differential expression of immunomodulatory genes and ROC curve analysis

**FIGURE 8:**
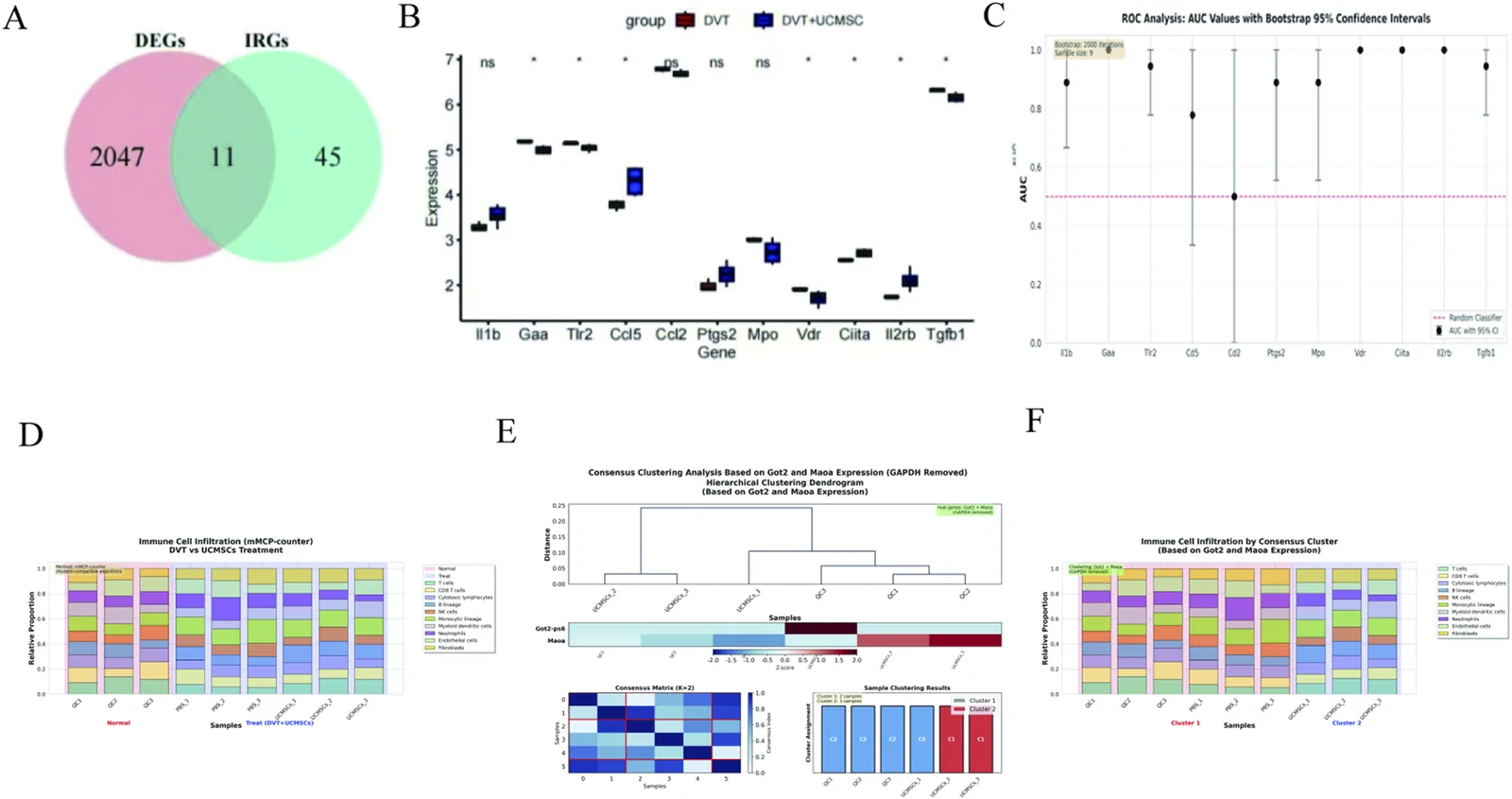
Validation of differential expression of immunomodulatory genes and immune infiltration analysis. **(A)** Venn diagram showing the intersections of DEGs and IRGs. **(B)** Expression profiles of 11 differentially expressed immunomodulatory genes in DVT and UC-MSCs samples. **(C)** ROC analysis of immune-related hub genes with bootstrap 95% confidence intervals. **(D)** Immune Cell Infiltration (mMCP-counter) in different groups (DVT, UC-MSCs) of the transcriptome dataset; **(E)** Consensus Clustering Analysis Based on Got2 and Maoa Expression (GAPDH Removed) Hierarchical Clustering Dendrogram; **(F)** Immune Cell Infiltration by Consensus Cluster (Based on Got2 and Maoa Expression).

Recent studies have demonstrated that immune cells are critically involved in the pathophysiology of DVT (Aklilu et al., 2025; Pekayvaz et al., 2025; Wang et al., 2024). Therefore, we systematically investigated the association between the core pathological characteristics of DVT and immune cell infiltration. To investigate the differential expression of immunomodulatory genes in the transcriptome dataset, we first identified the intersection between DEGs and immunoregulatory genes (IRGs), thereby obtaining a set of differentially expressed immunomodulatory genes (Figure 8A). Group comparison plots illustrated the expression profiles of 11 differentially expressed immunomodulatory genes in DVT and UC-MSCs samples. Statistical analysis revealed that the expression levels of seven key immunomodulatory genes—Gaa, Tlr2, Ccl5, Vdr, Ciita, Il2rb, and Tgfb1—differed significantly between DVT and UC-MSCs samples (*p* < 0.05) (Figure 8B). Among these seven genes, the immune regulatory genes Ccl5, Ciita, and Il2rb were upregulated following UC-MSCs treatment, while Gaa, Tlr2, Vdr, and Tgfb1 were downregulated, with significant differences in their expression levels. The list of the major upregulated and downregulated genes, encompassing gene names, log2 fold changes, p-values, is included in Supplementary Table 5. Their impact on the pathways analysis was shown in Supplementary Table 6. ROC analysis with bootstrap confidence intervals revealed that multiple immune-related genes demonstrated strong discriminatory ability between normal and treatment groups (Figure 8C; Supplementary Table 4). Four genes (Gaa, Vdr, Ciita, Il2rb) achieved perfect discrimination (AUC = 1.000, 95% CI: [1.000, 1.000]), indicating complete separation between groups based on expression levels. Additionally, Tlr2 and Tgfb1 showed excellent performance (AUC = 0.944, 95% CI: [0.778, 1.000]), while Il1b, Ptgs2, and Mpo demonstrated good discrimination (AUC = 0.889). The bootstrap confidence intervals confirm the robustness of these findings, with narrow CIs for the top-performing genes indicating stable and reliable discriminatory performance. We carefully position these results as exploratory findings.

### 3.7 Immune infiltration analysis

The transcriptome dataset was analyzed to estimate the abundance of infiltrating immune cells using the mMCP-counter algorithm. The proportions of 10 immune cell types in the DVT and UC-MSCs groups were visualized as bar charts (Figure 8D). To further investigate molecular subtypes among the treated samples, consensus clustering was performed with ConsensusClusterPlus based on the expression levels of two hub genes (Got2 and Maoa). This analysis identified two distinct subtypes (Figure 8E): subtype A (Cluster 1: DVT_1, DVT_3, UC-MSCs_2, DVT_2, UC-MSCs_1) and subtype B (Cluster 2: UC-MSCs_3). Subsequently, the mMCP-counter algorithm was applied to assess immune cell infiltration within each subtype, and the results were presented as bar charts (Figure 8F).

### 3.8 Safety of UC-MSCs treatment

**FIGURE 9:**
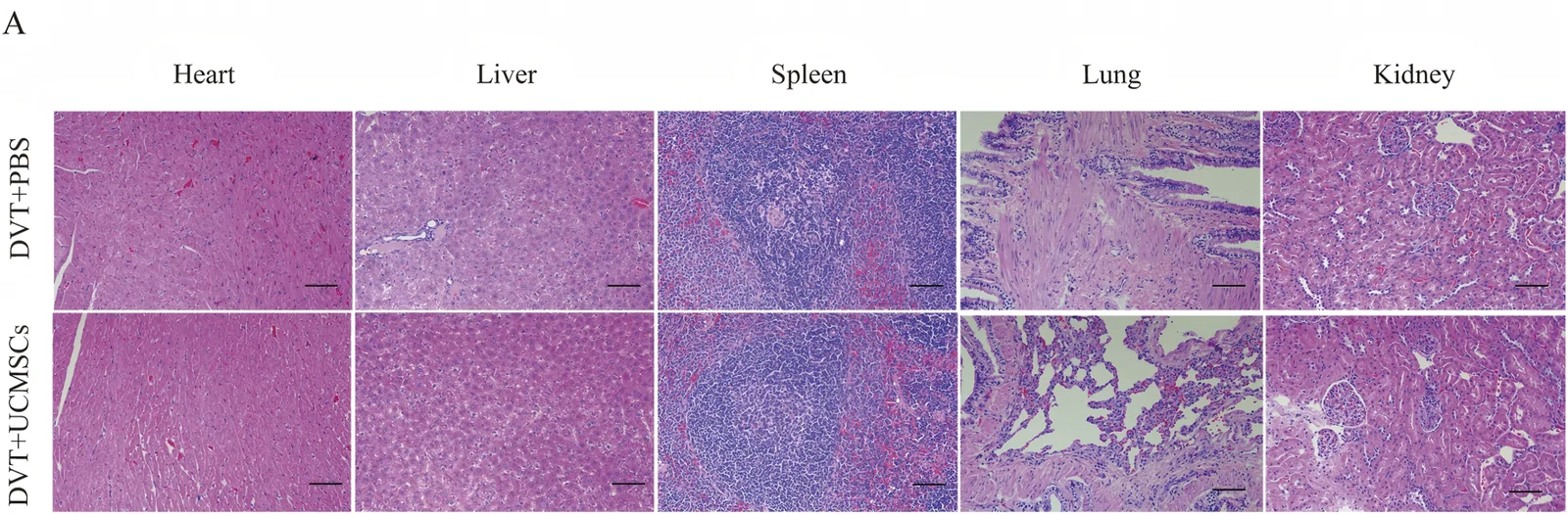
UC-MSCs treatment showed no toxicity in pregnant rats model. **(A)** HE staining of heart, liver, spleen, lungs, kidneys. Scale bars: 100 μm. No pathological changes such as tumors or thrombosis were observed.

This study comprehensively assessed the safety of UC-MSCs by histologically evaluating the major organs (heart, liver, spleen, lung, and kidney) of rats from all experimental groups using HE staining. As shown in Figure 9, the histological architecture of these organs in each group was consistent with previous findings (Cheng et al., 2023). Furthermore, blood biochemical markers indicative of hepatic and renal function, including ALT, AST, TBIL (including Direct Bilirubin and Indirect Bilirubin), CREA, UA, and UREA, were measured. All parameters remained within established normal reference ranges (Table 1). Collectively, these results demonstrate that UC-MSCs administration is safe for the treatment of DVT in pregnant subjects.

**TABLE 1:** Blood test indicators for liver and kidney function.

	sham+PBS	DVT+PBS	DVT+UC-MSCs	sham+UC-MSCs
ALT(U/L)	33.760	31.902	32.165	31.175
AST(U/L)	85.092	85.555	84.112	85.132
TBIL(umol/L)	1.412	1.524	1.598	1.218
Direct Bilirubin (umol/L)	0.613	0.732	0.563	0.449
Indirect Bilirubin(umol/L)	0.799	0.792	1.035	0.769
UREA(mmol/L)	6.127	6.802	6.659	5.896
UA(umol/L)	71.119	70.190	71.121	70.117
CREA(umol/L)	33.89	34.429	35.651	33.618

ALT, Alanine Aminotransferase; AST, Aspartate Aminotransferase; TBIL, Total Bilirubin; UREA, Urea; UA, Uric Acid; CREA, Creatinine. Data are expressed as mean ± standard deviation, n = 6. Normal reference range ofthe rat species: ALT(20-50U/L); AST(80-160U/L); TBIL(< 4.0 umol/L); Direct Bilirubin (< 1.5 umol/L); Indirect Bilirubin (< 2.5 umol/L); UREA(4.5-7.5 mmol/L); UA(70-150 umol/L); CREA(20-45 umol/L).

Source of the reference range: 《Handbook of Blood Physiology and Biochemistry for Laboratory Animals》.

To assess the safety of UC-MSCs, we conducted additional exams by performing HE staining on placental tissues from both the sham + PBS group and the DVT + UC-MSCs treatment group. The results indicated that the placental structures in both groups exhibited clarity and proper cellular shape. No placental harm, including vacuolarization or sinus reduction, was noted in the UC-MSCs therapy group. Histochemical examination of CD31 in placental tissues indicated that the brown ring-shaped lumen structures corresponded to CD31-positive placental microvessels, with no significant variations in microvascular density across the groups. Subsequent PCR analysis of vascular growth-related factors (VEGFA, PLGF, and sFlt-1) in placental tissues revealed no statistically significant alterations in vascular markers between the UC-MSCs treatment group and the control group. Furthermore, the weight assessment of placentas and fetuses revealed no significant disparities between the two groups (Supplementary Figure 7). According to initial safety results and our further data, we assess that “the risk of maternal-fetal transfer has not been detected in current PA-DVT therapies utilizing UC-MSCs.”

## 4 Discussion

PA-DVT represents a significant health threat due to its potential progression to PE, a life-threatening complication for pregnant women. Thrombus formation in the deep veins of the lower extremities can result in serious consequences for both mother and fetus (Alsheef et al., 2020; Cheng J. et al., 2022; Devis and Knuttinen, 2017). The pathophysiology of PA-DVT is complex, involving factors such as hormonal changes during gestation, venous stasis, and endothelial injury. These factors contribute to a hypercoagulable state, increasing the risk of thrombotic events in pregnant individuals. Consequently, maternal health is adversely affected, leading to substantial healthcare costs and social burdens (Havers-Borgersen et al., 2023; Zhou et al., 2024). The incidence of obstetric DVT ranges from 0.08% to 7.13% across studies, which is four to five times higher than in nonpregnant women of comparable age. Although the absolute incidence is low, DVT remains a leading cause of maternal mortality, accounting for 13.8% of maternal deaths in developed countries between 2003 and 2009 (Cheng J. et al., 2022; O'Shaughnessy et al., 2020).

The therapeutic efficacy of MSCs has traditionally been ascribed to their homing and guided differentiation capacities. Increasing data indicates that their therapeutic benefits predominantly depend on paracrine pathways (Raghav et al., 2022). The secretory function of extracellular vesicles, especially exosomes, is regarded as a vital element. Exosomes produced from MSCs exhibit therapeutic potential in numerous disease models due to their roles in immune regulation, tissue healing, angiogenesis stimulation, and drug delivery. In DVT, a complicated multifactorial pathological process, angiogenesis is essential for tissue repair and regeneration, significantly influencing thrombus organization, recanalization, and the functional recovery of damaged tissues. Consequently, we hypothesize that MSC-derived exosomes, with angiogenesis-promoting and other multifunctional attributes, may significantly contribute to the treatment of DVT.

To test this hypothesis, a PA-DVT model was established in SD rats to evaluate the therapeutic efficacy of UC-MSCs in this study. UC-MSCs treatment significantly reduced thrombus length and weight and promoted thrombus recanalization. Moreover, UC-MSCs improved pregnancy outcomes in rats with PA-DVT. *In vitro*, UC-CM attenuated hydrogen peroxide-induced endothelial cell injury. A significant issue is the therapeutic mechanism of human UC-MSCs under the distinctive immunological milieu of pregnancy. We propose that a distinct synergistic interaction occurs between the maternal immunological milieu and the immunomodulatory characteristics of human UC-MSCs. A successful pregnancy necessitates the maternal immune system to develop immunological tolerance towards the fetus. UC-MSCs, derived during the pregnancy process, exhibit elevated expression of many markers linked to pregnancy immunological tolerance, including indoleamine 2,3-dioxygenase (IDO), prostaglandin E2 (PGE2), and human leukocyte antigen G (HLA-G) (Ding et al., 2016; Soleymaninejadian et al., 2012). Consequently, exogenous human UC-MSCs may more efficiently assimilate into and augment the pre-existing immunological tolerance mechanisms in pregnant rats. Human UC-MSCs may provide therapeutic benefits by enhancing and facilitating pregnancy-specific immunological modifications, rather than merely initiating a generalized anti-inflammatory response. Collectively, these findings suggest that UC-MSCs may represent a promising therapeutic strategy for the management of PA-DVT. This work primarily examines the therapeutic effects of UC-MSCs, although the exosomes (MSC-EVs) generated from them, as potential therapeutic mechanisms and future avenues in stem cell therapy, warrant particular consideration.

A total of 2,058 DEGs associated with the pathological mechanisms of DVT were identified in this study. Elucidating the biological significance of these DEGs is critical for understanding the molecular processes underlying DVT development. GOT2 (Glutamic-Oxaloacetic Transaminase 2, also known as Aspartate Aminotransferase 2) is a key enzyme in amino acid transamination and central metabolic pathways, linking multiple important metabolites. Transcriptomic research revealed that, in comparison to the deep vein thrombosis (DVT) group, the expression of Got2 in the UC-MSCs group was markedly diminished. Western blot and PCR analysis further confirmed these findings. GOT2 expression may increase in response to cellular stressors, such as inflammation or hypoxia, which are implicated in thrombosis. Previous studies have suggested that GOT2 participates in metabolic and stress-related pathways that could indirectly affect coagulation processes (Wang et al., 2015). Elevated GOT2 activity during inflammation is typically indicative of endothelial cell injury or metabolic dysregulation (Hennigs et al., 2021). Subsequent endothelial secretion of von Willebrand factor (vWF) and tissue factor (TF) promotes coagulation factor activation. In summary, inflammation-driven upregulation of GOT2 contributes to endothelial dysfunction and hypercoagulability, both of which are mitigated following stem cell therapy. The complete designation of the MAOA molecule is Monoamine Oxidase A. Transcriptome analysis indicated that, in comparison to the DVT group, the expression level was elevated following treatment with UC-MSCs. Western blot and PCR studies corroborated these findings. MAOA may modulate the concentrations of inflammatory mediators and thereby influence the development of venous thrombosis. Cathcart et al. indicated that MAOA, *via* the oxidative destruction of biogenic amines (such as histamine and serotonin, which serve as inflammatory mediators), may induce naive monocytes to transition from a pro-inflammatory phenotype to an anti-inflammatory phenotype. MAOA diminishes the activation of inflammatory cells and the secretion of inflammatory substances, thereby reducing the likelihood of thrombosis development (Cathcart and Bhattacharjee, 2014).

Furthermore, our study revealed significant alterations in metabolite profiles following UC-MSCs therapy in the PA-DVT model. Metabolites with a VIP score greater than 1 were selected for further analysis. Notably, L-phenylalanine, a key component of the trans-metabolic pathway, was found to be associated with multiple critical genes. Previous research by Wang et al. demonstrated that L-phenylalanine preserves vascular function by enhancing BH4-mediated biosynthesis, reducing superoxide production by nitric oxide synthase, thereby decreasing reactive oxygen species (ROS) levels and increasing nitric oxide (NO) availability. As NO plays a protective role in maintaining the integrity of the HUVEC vascular wall, L-phenylalanine may indirectly inhibit thrombosis (Wang et al., 2021). In our pregnant rat DVT model, L-phenylalanine levels increased following UC-MSCs treatment, consistent with our metabolomic findings. Comprehensive analysis of these metabolic changes will not only enhance our understanding of DVT pathophysiology but also support the development of novel therapeutic strategies targeting these metabolic pathways.

Transcriptomic and metabolomic analyses have provided robust technical support for elucidating the protective effects of UC-MSCs in PA-DVT. KEGG pathway analysis revealed that DEGs and DMs regulated by UC-MSCs treatment are primarily enriched in phenylalanine metabolism and amino acid biosynthesis pathways. Obi et al. (2023) reported that older rats exhibited higher plasma concentrations of glutamine, phenylalanine, and proline compared to younger animals. These metabolites were correlated with vein wall weight and P-selectin levels, suggesting that age-related metabolic changes may contribute to the pathogenesis of venous thrombosis (Obi et al., 2016). Similarly, Maekawa et al. (2023) identified alterations in purine metabolism and phenylalanine concentrations in patients with DVT, indicating metabolic dysregulation within thrombus cells (Franczyk et al., 2021). Furthermore, Mendelian randomisation analysis has highlighted phenylalanine metabolism as a key pathway associated with DVT, and demonstrated that specific metabolites, such as phenylalanine, mediate the impact of gut microbiota on thrombotic events (Cheng et al., 2025). Jiang et al. conducted a metabolomic analysis of plasma samples from patients with acute cerebral venous thrombosis (CVT) and healthy controls, identifying 343 distinct compounds (Jiang et al., 2023). Notably, the caffeine metabolism pathway and the biosynthesis of branched-chain amino acids (BCAAs)—including valine, leucine, and isoleucine—were significantly altered in the CVT group compared to controls. In a review of metabolomic profiling in DVT and its chronic sequelae, Zahra et al. found substantial changes in amino acid, lipid, carbohydrate, and nucleotide metabolites. The tricarboxylic acid (TCA) cycle, biosynthesis of valine, leucine, and isoleucine, metabolism of alanine and aspartate, and D-glutamine and D-glutamate metabolism were among the most affected pathways in venous thrombosis (Amirsardari et al., 2025).

Meanwhile, Amino acids are the fundamental building blocks of proteins, and robust protein synthesis is essential for angiogenesis. The amino acid biosynthetic pathway ensures that vascular endothelial cells and other relevant cell types obtain sufficient amino acids, thereby supporting protein synthesis and promoting angiogenic processes. Certain enzymes and metabolites within the phenylalanine metabolic pathway participate in intracellular signal transduction and modulate gene expression. These genes, such as vascular endothelial growth factor (VEGF) and its receptors, are closely associated with angiogenesis. Regulation of these genes *via* the phenylalanine metabolism pathway can influence both the initiation and progression of angiogenesis. The findings indicate that metabolites and essential enzymes related to phenylalanine metabolism and amino acid biosynthesis may affect angiogenesis and thrombus development during UC-MSCs therapy for PA-DVT, presenting new targets for future thrombosis prevention and treatment efforts.

MicroRNAs (miRNAs) are extensively involved in cellular physiological and pathological processes *via* epigenetic regulation of gene expression, a phenomenon that has garnered considerable attention in the academic community (Nalbant and Akkaya-Ulum, 2024; Yang et al., 2024). Increasing evidence indicates that miRNAs play a significant role in DVT (Gareev et al., 2023; Luo et al., 2025; Zhang D. et al., 2024). Building on these findings, we predicted miRNAs associated with two hub genes—Maoa and Got2—and identified mRNA–miRNA pairs that were present in multiple databases and exhibited high connectivity, thereby constructing an mRNA–miRNA regulatory network. Furthermore, we investigated the regulatory relationships between transcription factors and these two core genes. Notably, transcription factors such as KLF4, YY1, SP1, and MYC are regulated by Maoa and Got2.

Recent studies have increasingly elucidated the pivotal role of immune cells in the pathophysiology of DVT (Aklilu et al., 2025; Pekayvaz et al., 2025; Wang et al., 2024). Inflammation and immune responses are closely associated with the development of venous thrombosis (Najem et al., 2020; Otasevic et al., 2022). Immune cells and inflammatory mediators play pivotal roles in thrombus formation, particularly during the repair and regeneration phases following venous thrombosis (Aksu et al., 2012; Vazquez-Garza et al., 2017). Notably, monocytes and lymphocytes can regulate the coagulation cascade, while coagulation products, in turn, modulate immune responses. Motivated by these findings, we systematically explored the potential association between the therapeutic mechanism of UC-MSCs in PA-DVT and immune cell infiltration. Comparative transcriptomic analysis of DVT and UC-MSCs-treated samples identified seven immune regulatory genes with significant differential expression (Figure 8B), including Gaa, Tlr2, Ccl5, Vdr, Ciita, Il2rb, and Tgfb1. These differentially expressed immune regulatory genes may play a critical role in the therapeutic mechanism of UC-MSCs for DVT during pregnancy. Our ROC analysis, strengthened by bootstrap confidence interval estimation, identified several immune-related genes with robust discriminatory ability for distinguishing between normal and DVT-affected samples. The perfect discrimination achieved by Gaa, Vdr, Ciita, and Il2rb (AUC = 1.000 with narrow bootstrap CIs) suggests these genes may serve as potential biomarkers for disease status. The use of stratified bootstrap resampling (2,000 iterations) ensures that our AUC estimates appropriately account for sampling variability and provides transparent reporting of statistical uncertainty. These findings align with the known roles of these genes in immune regulation and inflammatory responses, further supporting their biological relevance in DVT pathogenesis. Collectively, these findings suggest that the identified genes not only serve as potential biomarkers for UC-MSCss-mediated modulation of DVT during pregnancy but also represent promising targets for early diagnosis and therapeutic intervention.

This study possesses certain drawbacks. The KEGG enrichment analysis of metabolomics and transcriptomics indicated that the therapeutic benefits of UC-MSCs mostly occur *via* the pathways of amino acid biosynthesis and phenylalanine metabolism. Despite identifying multiple enriched pathways, the constraints of the study’s scope precluded comprehensive research into all routes. The therapeutic actions of UC-MSCs may also engage additional pathways, which we intend to investigate in forthcoming investigations. Secondly, in subsequent research, we will investigate extracellular vesicles, cytokine panels, or fractionation to link the bioactivity of UC-CM with *in-vivo* efficacy. Third, our research employed a SD pregnant rat model. While we have identified possible treatment targets, it remains uncertain if these findings are relevant to humans. Subsequent research should further corroborate the biological relevance of these findings in both *in vivo* and *in vitro* contexts. Fourth, while this study focuses on the therapeutic effects of UC-MSCs, MSC-EVs as a potential mechanism and future research direction deserve attention. Fifth, a limitation of this study is the absence of *a priori* power analysis to determine the required animal sample size. Future research should conduct rigorous sample size calculations based on the effect sizes obtained from this preliminary study before initiating experiments, ensuring statistically significant results. Sixth, future study may incorporate high-resolution imaging techniques (e.g., Doppler) to enhance the thrombosis recanalization assessment system.

In summary, this study systematically investigated the therapeutic effects and underlying mechanisms of UC-MSCs on DVT in pregnant rats using integrated metabolomics, transcriptomics, and molecular biology approaches. The results demonstrated that UC-MSCs treatment significantly reduced thrombus length and weight, promoted thrombus recanalization, and improved pregnancy outcomes in DVT-affected rats. By combining bioinformatics analyses with experimental validation, this research provides a comprehensive understanding of the therapeutic potential of UC-MSCs for PA-DVT. Notably, the findings reveal key metabolic and transcriptional pathways involved in the treatment process and identify relevant therapeutic targets. Overall, this work underscores the value of multi-omics strategies in elucidating the complex pathophysiology of DVT during pregnancy and offers novel perspectives for the development of targeted therapies.

## Data Availability

The datasets presented in this study can be found in online repositories. The names of the repository/repositories and accession number(s) can be found in the article/Supplementary Material.
