# Correction: Elucidating the protective mechanisms of umbilical cord mesenchymal stem cells against stenosis-induced deep venous thrombosis during pregnancy: a transcriptomic and metabolomic study

**DOI:** 10.3389/fcell.2026.2010754

**Published:** 2026-10-30

**Authors:** Junrong Zhang, Feng Sun, Jingjing Yao, Jianlin Zhang, Xirong Wu, Yunzhao Xu, Yuquan Zhang, Xi Cheng

**Affiliations:** 1 Department of Gynecology and Obstetrics, Affiliated Hospital of Nantong University, Medical School of Nantong University, Nantong, China; 2 Trauma Center, Affiliated Hospital of Nantong University, Nantong, China

**Keywords:** immune regulatory genes and metabolites, metabolomics, obstetric DVT, transcriptomics, UC-MSCs

There was a mistake in Figure 4A as published. During figure preparation, an incorrect representative image was inadvertently inserted for the 12 h Conditional media group in Figure 4A. After re-examining the original experimental image files, we confirmed that this error occurred during figure assembly. The incorrect representative image has been replaced with the correct image from the original experiment. The corrected Figure 4 appears below.

**FIGURE 4:**
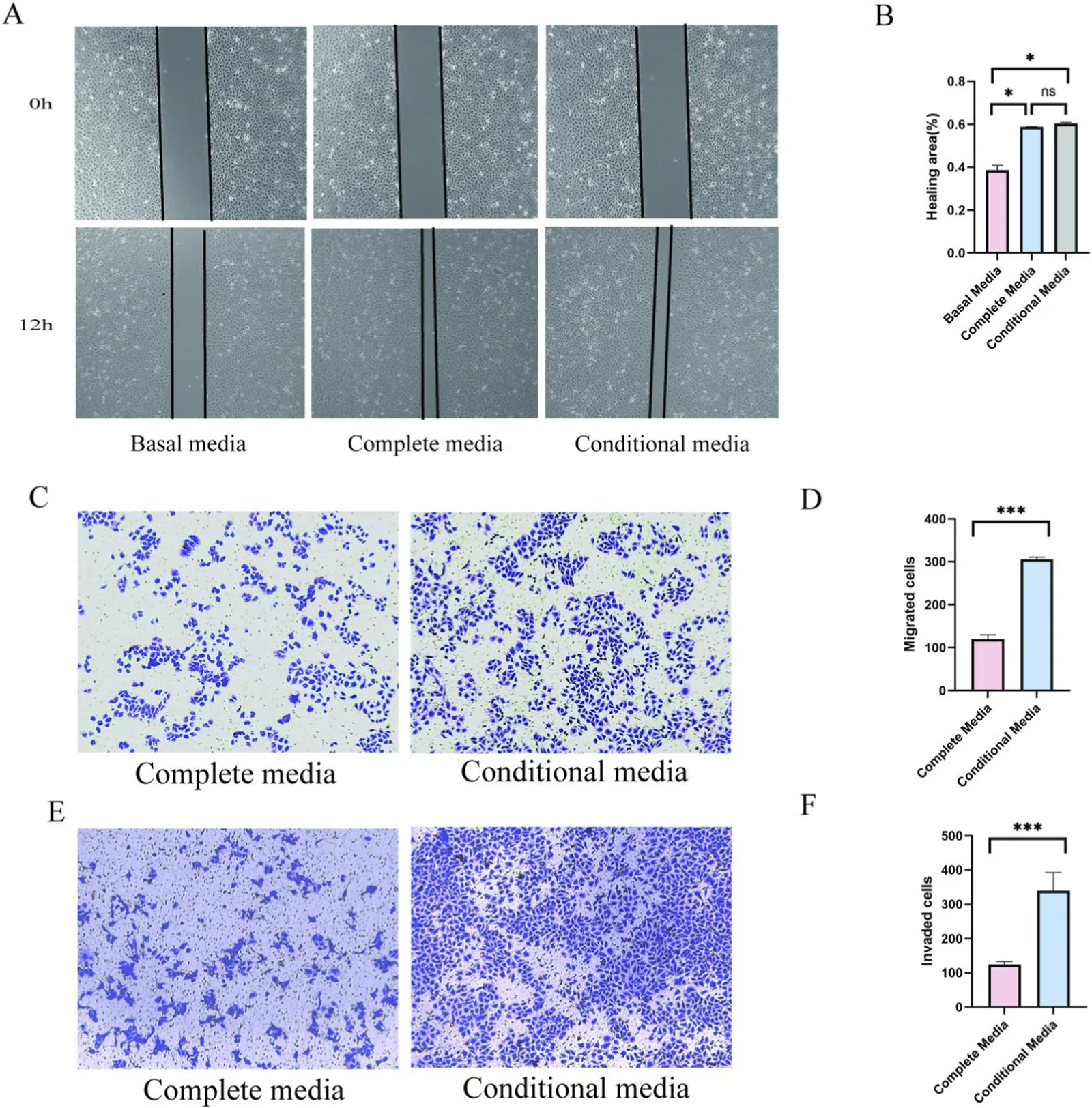
UC-CM can repair endothelial cell damage induced by H_2_O_2_. **(A)** The effect of UC-CM on the healing of damaged endothelial cells was assessed using the scratch assay. Scale bars: 100 μm; **(B)** Statistical analysis results of healing area; **(C, D)** Numbers of crystal violet-stained migrated cells. Scale bars: 100 μm; **(E, F)** Numbers of crystal violet-stained invased cells. Scale bars: 100 µm. (n = 3 biological replicates). Data are presented as the mean ± SEM. (*p < 0.05, **p < 0.01, ***p < 0.001, ns, no significance).

This correction does not affect the quantitative analyses, statistical results, interpretation of the data, or the conclusions of the article.

The authors sincerely apologize for this error and for any confusion or inconvenience it may have caused.

The original version of this article has been updated.

