# Supplementary material for: Elucidating the protective mechanisms of umbilical cord mesenchymal stem cells against stenosis-induced deep venous thrombosis during pregnancy: a transcriptomic and metabolomic study
Source: Front Cell Dev Biol. 2026 Jan 12;13:1690377. doi: 10.3389/fcell.2025.1690377 (PMC12832865; doi:10.3389/fcell.2025.1690377)
Supplement: Supplementary file 1 [file Supplementaryfile1.zip › Supplementary files/Supplementary file.docx]

**Elucidating the protective mechanisms of umbilical cord mesenchymal stem cells against stenosis-induced deep venous thrombosis during pregnancy: A transcriptomic and metabolomic study**

**Running title: Protective mechanisms of UC-MSCs against PA-DVT**

Junrong Zhang ^1†^, Feng Sun ^2†^, Jingjing Yao ^1†^, Jianlin Zhang ^1^, Xirong Wu ^1^, Yunzhao Xu ^1^, Yuquan Zhang ^1, *^, Xi Cheng ^1, *^

^1^ Department of Gynecology and Obstetrics, Affiliated Hospital of Nantong University, Medical School of Nantong University, Nantong 226001, China;

^2^ Trauma Center, Affiliated Hospital of Nantong University, Nantong 226001, China

Junrong Zhang, Feng Sun and Jingjing Yao contributed equally to this study.

^*^Corresponding author.

E-mail addresses: [jsnt_zhangyuquan@163.com](mailto:jsnt_zhangyuquan@163.com) (Y. Zhang), [1375072047@qq.com](mailto:jsnt_zhangyuquan@163.com) (X. Cheng).

**2 Materials and methods**

**2.9 Western blotting**

**Information of antibodies.** Endothelial nitric oxide synthase (eNOS, 1:1000; ABclonal), Thrombomodulin (TM, 1:5000; Proteintech), (VEGFA, 1:2000; Proteintech).

**2.10 RT-qPCR assay**

The primer sequences for PLGF, sFlt1 and VEGFA were as follows: PLGF in rat (F: cca actcgtccctgctgaatgac; R: ggaaccgtggctggcttcttc), sFlt1 in rat (F: gagcatctatcaggcagcggattg; R: cga cccactcttcacacgacaag), VEGFA in rat (F: cggtgtggtctttcgtcctcttag; R: agggatgggtttgtcgtgttctg).

**2.13 Non-targeted metabolomics profiling**

Metabolite extraction was performed as detailed below: the sample was extracted from the -80℃ freezer and gradually thawed at 4℃. Obtain a suitable sample and include it into a pre-chilled internal standard solution of MeOH:ACN:H2O (v:v:v=2:2:1), accompanied by two steel beads. Process the combination in a tissue grinder at 60Hz for 120 seconds, then subject it to ultrasonic treatment for 10 minutes. Incubate at -20℃ for 1 hour, followed by centrifugation at 13,000 rpm for 15 minutes at 4℃; collect the supernatant and lyophilize. In mass spectrometry, introduce a suitable volume of ACN:H2O solution (v:v=1:1) for resolubilization, followed by vortexing for 30 seconds and ultrasonication for 10 minutes. Centrifuge at 13,000 rpm for 15 minutes at 4°C; transfer the supernatant into a sample vial for LC-MS/MS analysis. Furthermore, 10µl of each sample is combined to create a QC sample, which is subsequently bottled.

A pooled quality control (QC) sample was utilized to check instrumental stability and guarantee data quality during the acquisition sequence. The QC sample was created by amalgamating equal quantities (10 μL) from each unique experimental sample across all groups, thus encapsulating the complete chemical composition spectrum of the investigation. The pooled quality control was administered at consistent intervals (e.g., after every 6-8 experimental samples) during the analytical procedure.

**2.14 Difference analysis**

To guarantee data integrity for subsequent statistical analysis, we instituted a filtering procedure focused on metabolite attributes exhibiting significant technical variability. We computed the relative standard deviation (RSD) for each metabolite characteristic in the quality control samples, excluding those with an RSD more than 30%. This filtering process successfully eliminates unstable signals that are significantly influenced by technical noise and instrument drift, hence improving the reliability of subsequent multivariate statistical analyses and biomarker screening outcomes. This technique primarily functions as a data "filtering" mechanism rather than a direct "correction" of signal drift. Future research will utilize specific methods or software for enhanced active drift correction to further elevate data quality. The data filtering phase is finalized prior to the subsequent differential metabolite screening, which relies on VIP values over 1 and p-values below 0.05, serving as a crucial prerequisite for constructing a high-quality dataset.

**2.18 Immune-Related Gene ROC Analysis**

### **1. Bootstrap Confidence Interval Analysis**

We performed **stratified bootstrap resampling** (2,000 iterations) to estimate 95% confidence intervals (CIs) for the AUC of each immune-related gene. This approach is widely recognized as the gold standard for assessing uncertainty in diagnostic performance metrics, particularly when sample sizes are limited (Efron & Tibshirani, 1993; Steyerberg et al., 2001).

**Methodology:** - **Algorithm:** Stratified bootstrap with 2,000 iterations - **Sampling strategy:** Independent resampling with replacement from each class, maintaining the original class proportions - **CI calculation:** Percentile method (2.5th and 97.5th percentiles of bootstrap AUC distribution) - **Implementation:** Custom R script using the pROC package with stratified sampling to ensure balanced representation.

**Advantages of stratified bootstrap:** - Maintains class balance in each bootstrap sample - Provides stable CI estimates even with modest sample sizes - Accounts for sampling variability without requiring data splitting - More appropriate than cross-validation for very small datasets (Vabalas et al., 2019)
