# Supplementary material for: Elucidating the protective mechanisms of umbilical cord mesenchymal stem cells against stenosis-induced deep venous thrombosis during pregnancy: a transcriptomic and metabolomic study
Source: Front Cell Dev Biol. 2026 Jan 12;13:1690377. doi: 10.3389/fcell.2025.1690377 (PMC12832865; doi:10.3389/fcell.2025.1690377)
Supplement: Supplementary file 1 [file Supplementaryfile1.zip › Supplementary Table/Supplementary Table 1.docx]

| Number | preparations | Test items | Detection method | Test results |
| --- | --- | --- | --- | --- |
|  |  | Sterile  Mycoplasma  HIV | *CHP(2015) 1101*  *CHP(2015) 3301*  *Quantify PCR* | Negative  Negative  Qualified |
|  | 1, 2, 3 | HBV  HCV  TP  Bacterial endotoxin  Bovine serum residue | *Quantify PCR*  *Quantify PCR*  *Quantify PCR*  *Gel method*  *CHP(2015) 3411* | Qualified  Qualified  Qualified  < 2 EU·mL -1  < 30 mg ·L -1 |
