# Supplementary material for: Elucidating the protective mechanisms of umbilical cord mesenchymal stem cells against stenosis-induced deep venous thrombosis during pregnancy: a transcriptomic and metabolomic study
Source: Front Cell Dev Biol. 2026 Jan 12;13:1690377. doi: 10.3389/fcell.2025.1690377 (PMC12832865; doi:10.3389/fcell.2025.1690377)
Supplement: Supplementary file 1 [file Supplementaryfile1.zip › Supplementary Table/Supplementary Table 4.docx]

Supplementary Table 4 Bootstrap analysis of eleven genes

| Gene | AUC | 95% CI | SE | Interpretation |
| --- | --- | --- | --- | --- |
| Gaa | 1.000 | [1.000, 1.000] | 0.000 | Perfect discrimination |
| Vdr | 1.000 | [1.000, 1.000] | 0.000 | Perfect discrimination |
| Ciita | 1.000 | [1.000, 1.000] | 0.000 | Perfect discrimination |
| II2rb | 1.000 | [1.000, 1.000] | 0.000 | Perfect discrimination |
| Tlr2 | 0.944 | [0.778, 1.000] | 0.167 | Excellent discrimination |
| Tgfb1 | 0.944 | [0.778, 1.000] | 0.167 | Excellent discrimination |
| II1b | 0.889 | [0.667, 1.000] | 0.222 | Good discrimination |
| Ptgs2 | 0.889 | [0.556, 1.000] | 0.333 | Good discrimination |
| Mpo | 0.889 | [0.556, 1.000] | 0.333 | Good discrimination |
| Ccl5 | 0.778 | [0.333, 1.000] | 0.444 | Moderate discrimination |
| Ccl2 | 0.500 | [0.000, 1.000] | 0.500 | No discrimination |

*Note: For genes with original AUC < 0.5, we report 1-AUC to reflect reverse prediction (i.e., lower expression predicts the positive class), consistent with standard ROC analysis practice.*
