# Supplementary figures and images for: Elucidating the protective mechanisms of umbilical cord mesenchymal stem cells against stenosis-induced deep venous thrombosis during pregnancy: a transcriptomic and metabolomic study
Source: Front Cell Dev Biol. 2026 Jan 12;13:1690377. doi: 10.3389/fcell.2025.1690377 (PMC12832865; doi:10.3389/fcell.2025.1690377)

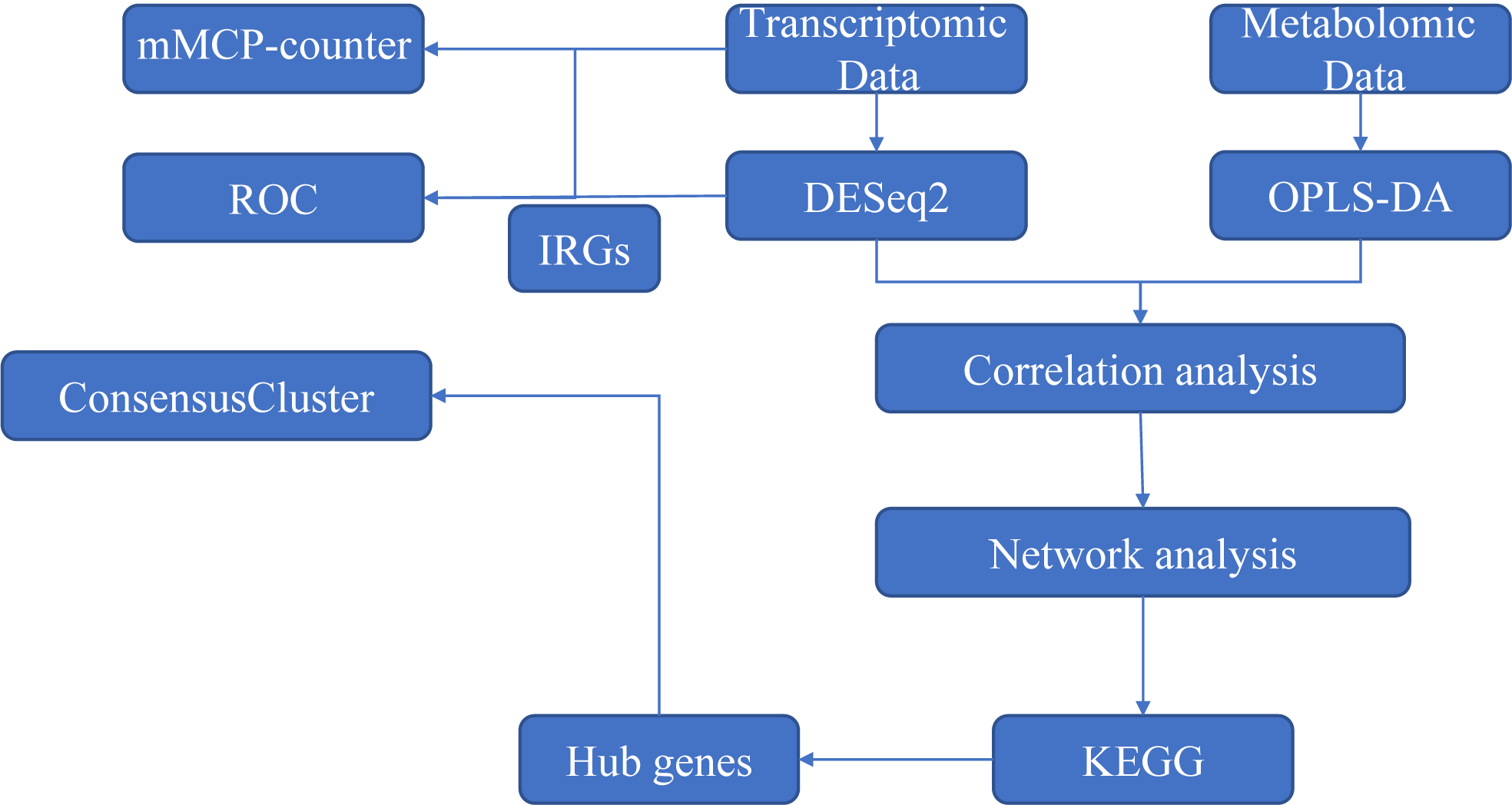

Supplement: Supplementary file 1 [file Supplementaryfile1.zip › Supplementary Figure/Supplementary Figure 1.tif]

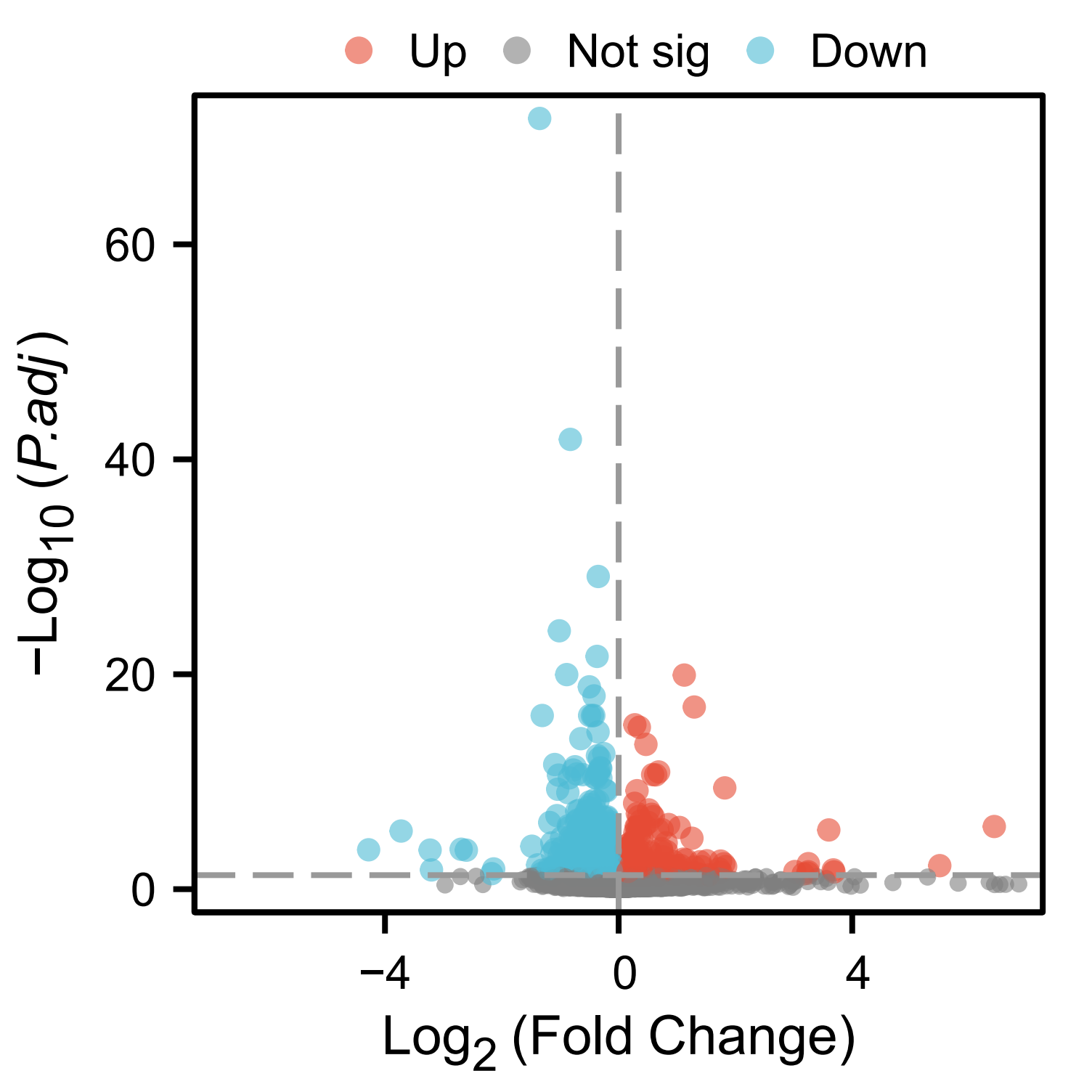

Supplement: Supplementary file 1 [file Supplementaryfile1.zip › Supplementary Figure/Supplementary Figure 2.tif]

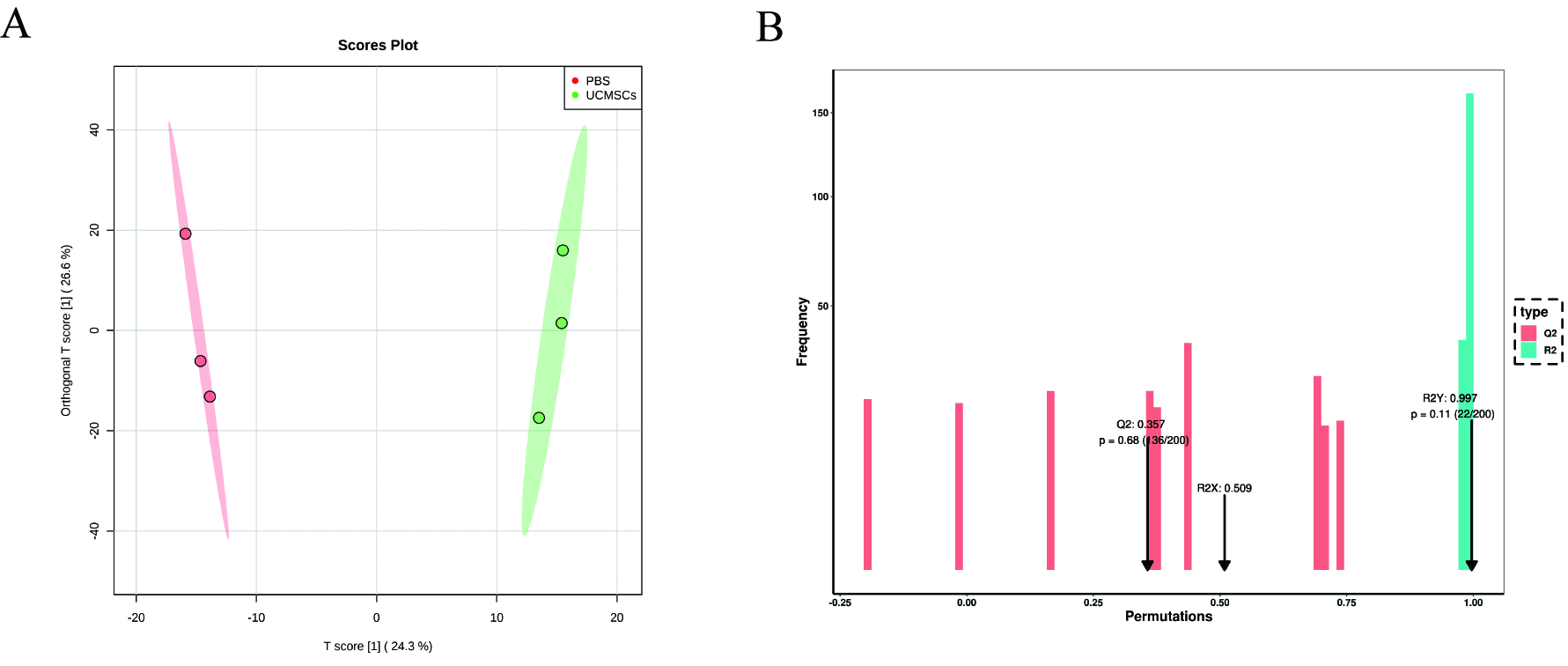

Supplement: Supplementary file 1 [file Supplementaryfile1.zip › Supplementary Figure/Supplementary Figure 3.tif]

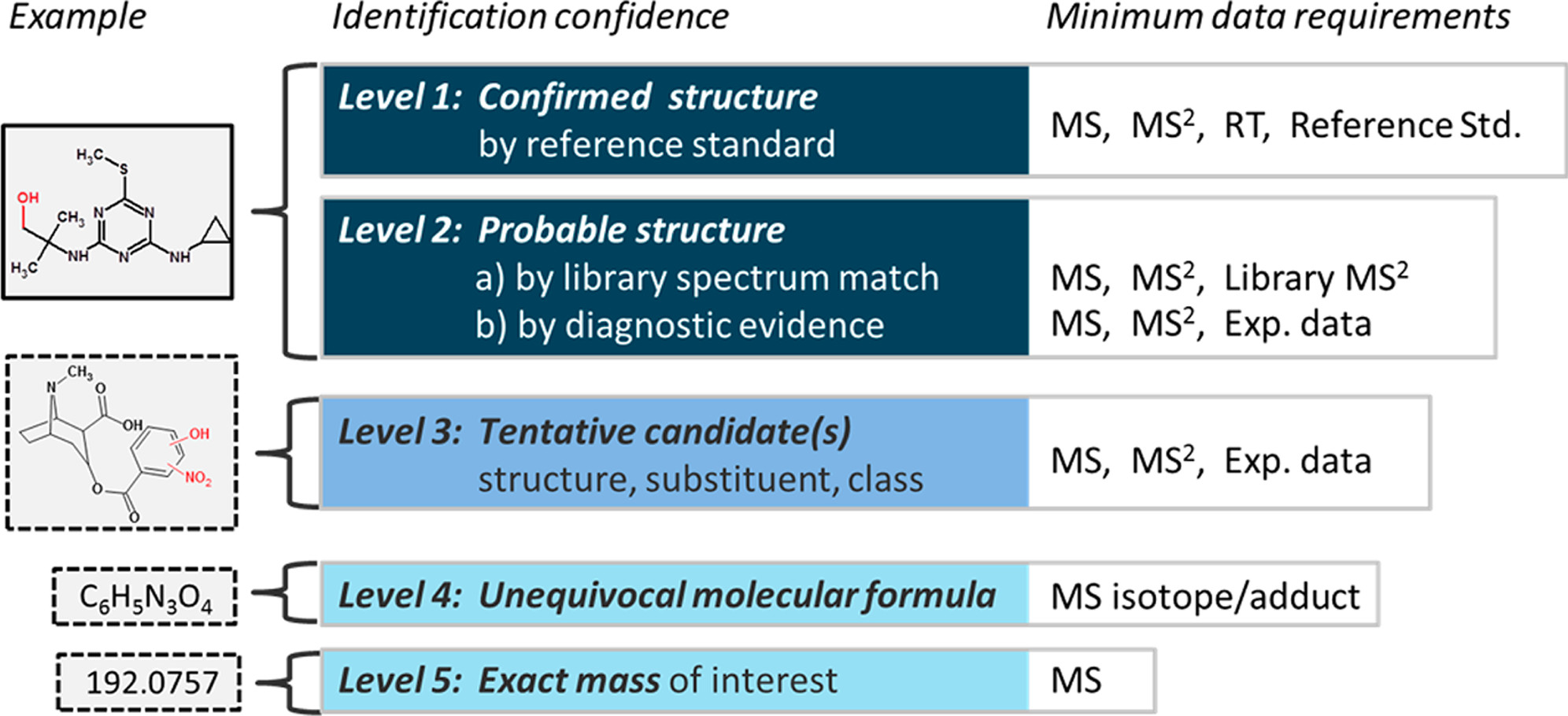

Supplement: Supplementary file 1 [file Supplementaryfile1.zip › Supplementary Figure/Supplementary Figure 4.tif]

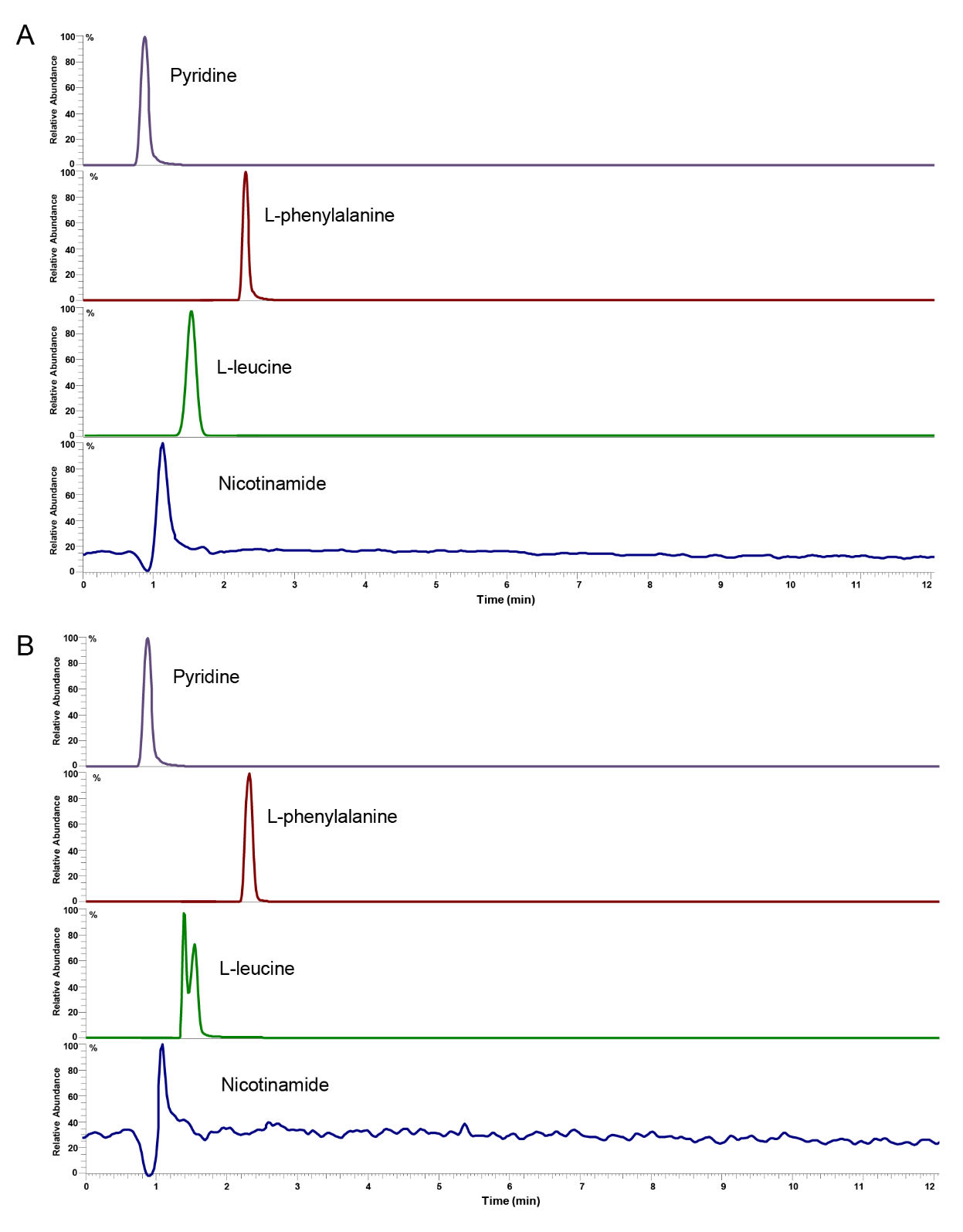

Supplement: Supplementary file 1 [file Supplementaryfile1.zip › Supplementary Figure/Supplementary Figure 5.tif]

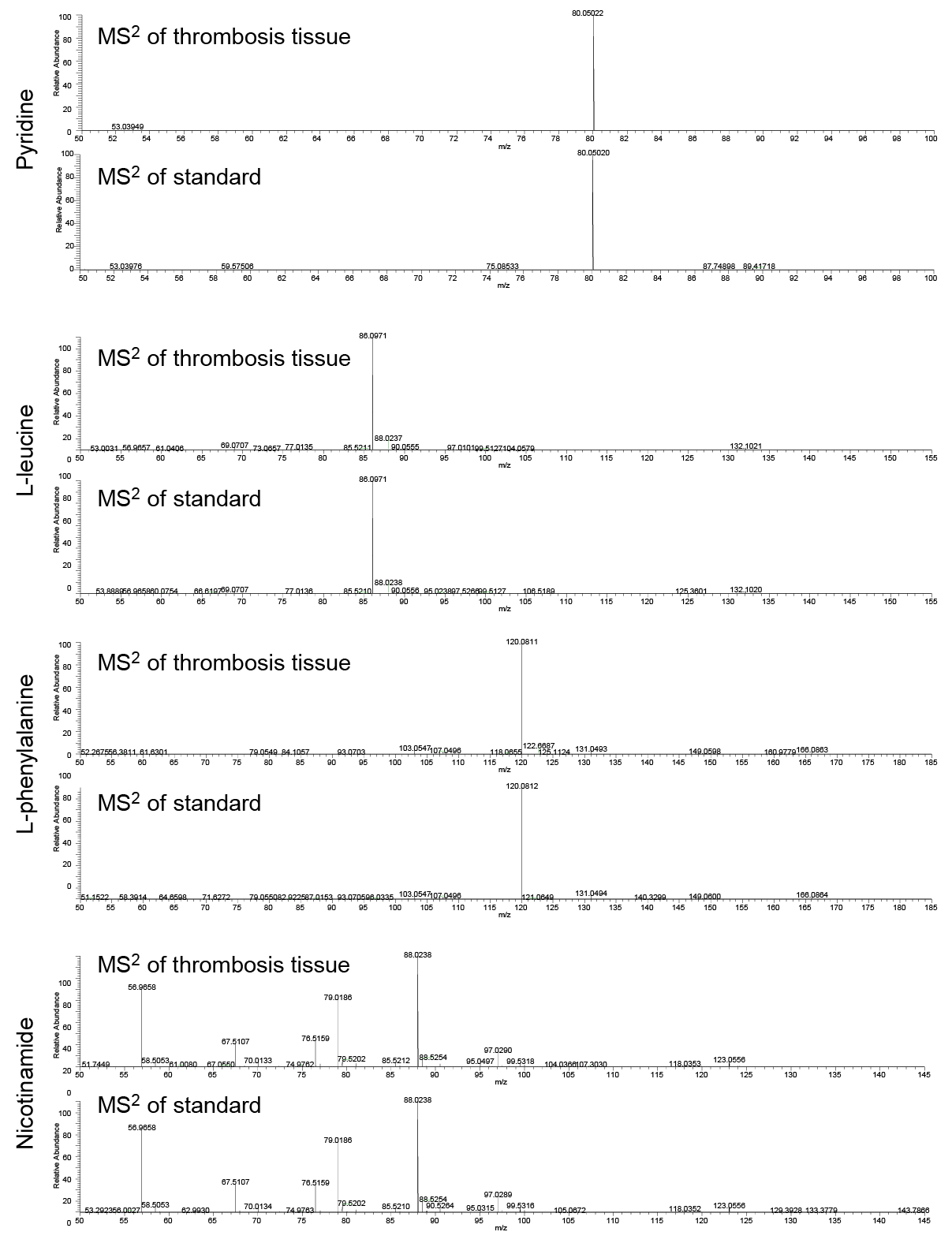

Supplement: Supplementary file 1 [file Supplementaryfile1.zip › Supplementary Figure/Supplementary Figure 6.tif]

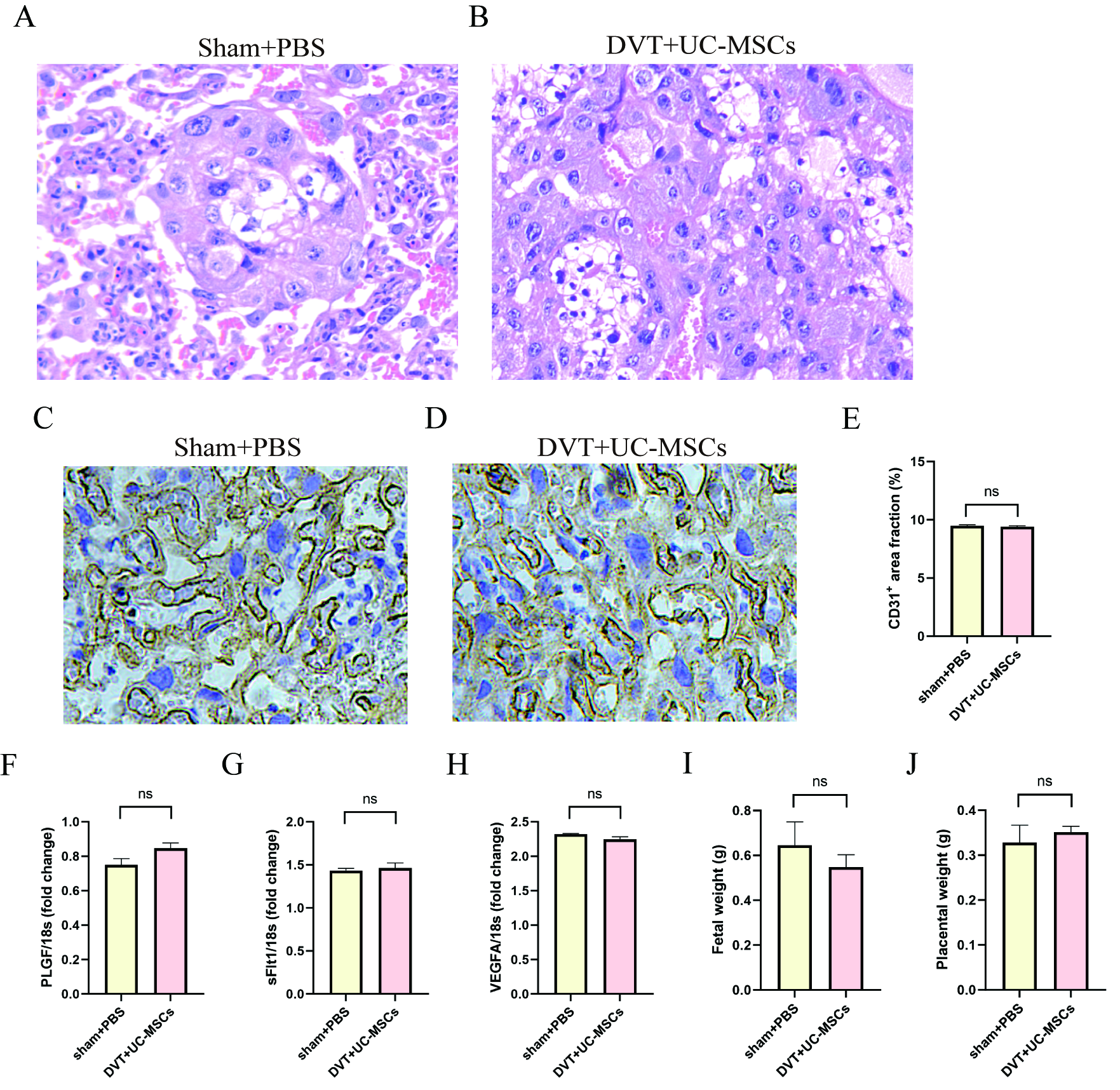

Supplement: Supplementary file 1 [file Supplementaryfile1.zip › Supplementary Figure/Supplementary Figure 7.tif]
