# Supplementary material for: Elucidating the protective mechanisms of umbilical cord mesenchymal stem cells against stenosis-induced deep venous thrombosis during pregnancy: a transcriptomic and metabolomic study
Source: Front Cell Dev Biol. 2026 Jan 12;13:1690377. doi: 10.3389/fcell.2025.1690377 (PMC12832865; doi:10.3389/fcell.2025.1690377)
Supplement: Supplementary file 1 [file Supplementaryfile1.zip › Supplementary files/Repeated independent experiments in vitro.doc]

Figure 3A-scratch assay (Basal media)-0h:

Figure 3A-scratch assay (Basal media)-12h:

Figure 3A-scratch assay (Complete media)-0h:

Figure 3A-scratch assay (Complete media)-12h:

Figure 3A-scratch assay (Conditional media)-0h:

Figure 3A-scratch assay (Conditional media)-12h:

Figure 3C-Complete media：

Figure 3C-Conditional media：

Figure 3E-Complete media：

Figure 3E-Conditional media：
